# A systematic review of active group-based dance, singing, music therapy and theatrical interventions for quality of life, functional communication, speech, motor function and cognitive status in people with Parkinson’s disease

**DOI:** 10.1186/s12883-020-01938-3

**Published:** 2020-10-10

**Authors:** Maxwell S. Barnish, Susannah M. Barran

**Affiliations:** 1 Peninsula Technology Assessment Group (PenTAG), Institute of Health Research, University of Exeter Medical School, Exeter, UK; 2 Evidence Synthesis and Modelling for Health Improvement (ESMI), University of Exeter Medical School, Exeter, UK; 3 Children and Young People’s Speech and Language Therapy, Evelina London Community Children’s Services, Mary Sheridan Health Centre, Guy’s and St Thomas’ NHS Foundation Trust, London, UK

**Keywords:** Parkinson’s disease, Singing, Music, Dance, Theatre, Systematic review

## Abstract

**Background:**

Parkinson’s disease (PD) is a common neurodegenerative condition associated with a wide range of motor and non-motor symptoms. There has been increasing interest in the potential benefit of performing arts as a therapeutic medium in PD. While there have been previous reviews, none have considered all performing arts modalities and most have focused on dance. This systematic review examined the potential benefit of all active group-based performing arts interventions for quality of life, functional communication, speech, motor function and cognitive status.

**Methods:**

Searches were conducted in February 2020 on five scholarly databases. Supplementary searches were conducted. Included studies were quantitative in design, and assessed the potential benefit of any active group-based performing arts intervention for quality of life, functional communication, speech, motor function or cognitive status in people with PD. Full text papers were eligible for inclusion, as were conference abstracts since January 2018. Screening, data extraction, narrative synthesis and quality assessment were conducted independently by two reviewers. Quality assessment used the SURE checklists.

**Results:**

Fifty-six studies were eligible for inclusion in this systematic review, reported in 67 publications. Published from 1989 to 2020, these studies included a total of 1531 people with PD from 12 countries, and covered four broad performing arts modalities: dance, singing, music therapy and theatre. Dance remains the most commonly studied performing arts modality for PD (38 studies), while there were 12 studies on singing interventions, four on music therapy, and only two on theatrical interventions. There was evidence for a beneficial effect of all four performing arts modalities on at least some outcome domains.

**Conclusions:**

This is the first systematic review to assess the potential benefit of all active group-based performing arts interventions in PD. The evidence suggests that performing arts may be a useful therapeutic medium in PD. However, a substantial limitation of the evidence base is that no studies compared interventions from different performing arts modalities. Moreover, not all performing arts modalities were assessed for all outcome domains. Therefore it is not currently possible to determine which performing arts modalities are most beneficial for which specific outcomes.

## Background

### Parkinson’s disease

Parkinson’s disease (PD) is an age-related neurodegenerative condition that can affect movement and motor control [1] as well as present with a wide range of non-motor symptoms [2] including cognitive impairment. Speech and communication difficulties are common in PD [3] and may result from a combination of motor and non-motor factors [4]. PD is one of the most common neurodegenerative conditions with 6.1 million people worldwide estimated to have PD in 2016 [5] with the prevalence expected to rise with the global ageing population [6]. The precise aetiology of PD remains unknown. However, it is known that the pathogenesis of PD, in particular cognitive functioning, involves the cholinergic, [7] serotonergic [8] and noradrenergic [9] systems, as well as the dopamine system which was historically seen as the sole system implicated in PD neuropathology [10]. The impact of PD on the quality of life of individuals can be substantial [11, 12]. Moreover, there can be substantial caregiver burden, [13] which may result in part from the combination of motor and non-motor symptoms and from communication difficulties. There is also a high social and economic impact of PD: for example, between 1994 and 2013 the mean annual health care cost difference in the UK between individuals with PD and controls was £2471 per annum per person at 2013 costs, increasing to £4004 10 years after diagnosis [14].

### Treatments for Parkinson’s disease

In recent decades, levodopa-based pharmacotherapy has become the mainstay of treatment for PD, demonstrating good efficacy in targeting motor symptomatology [15, 16]. However, dyskinesia – impairment of voluntary movement – as a side-effect is a substantial drawback to this treatment regimen [17]. Also, there is a lack of reliable evidence of benefit on speech [18, 19] and non-motor symptoms including cognition. These limitations of available pharmacotherapy regimens have led to increasing interest in non-pharmacological interventions in PD. There has been interest in how a wide range of allied health approaches may help address a wider range of PD symptoms. Exercise is a therapeutic modality that may be effective for PD, including for cognitive symptoms [20]. Speech and language therapy (SLT), in various forms, has been a popular approach to seek to address the speech and communication difficulties associated with PD. However, the robustness of the evidence base for current SLT approaches in PD remains problematic with one structured narrative review [20] and one Cochrane systematic review [21] finding no reliable evidence of significant benefit, and one systematic review [22] finding some evidence of benefit in limited areas of motoric speech production. The potential challenges facing SLT in PD are multifaceted, but one challenge identified in the United Kingdom is a traditional tendency for therapeutic techniques to focus largely on motoric aspects of speech production at the expense of functional communication, [23] whereas people with PD have reported social isolation resulting from functional communication difficulties to be much more burdensome than motoric speech impairment, [24] suggesting a misalignment of priorities. It should be noted that SLT approaches to PD differ internationally with the Lee Silverman Voice Therapy method [25] being standard clinical practice in some health systems. The difficulties encountered by SLT in PD have led to increased interest in a broader range of interventions that may provide benefit to different aspects of the PD symptom profile, including through everyday activities. Since social isolation plays such a key role in driving poor quality of life in PD – social isolation is for example a domain of the Nottingham Health Profile [26] and difficulties in this domain were reported by the majority of people with PD [27] – a broader range of interventions that increase social contact may be beneficial.

### Performing arts as therapy for Parkinson’s disease

The performing arts are a therapeutic modality in which there has been increasing interest, including in neurological conditions such as PD. Participation in the performing arts has been demonstrated to bring a range of psychosocial and health-related benefits in the general population as well as a wide range of diseases [28]. The identified benefits of performing arts can be broadly subdivided into instrumental and intrinsic benefits. Identified instrumental benefits of the performing arts include cognitive, attitudinal and behavioural and health benefits at the individual level as well as social and economic benefits at the community level. Additionally, the arts broaden and deepen an individual’s understanding of the world, [29] while there is evidence [30] that choral singing may offer social and mental health benefits to disadvantaged adults at personal, social and functional levels. One interesting aspect of the findings is how forming a new group identity as a choir member was associated with emotional and health benefits, consistent with social identity theory [31]. The intrinsic benefits of the arts [28] relate to ways in which effects intrinsic in the arts experience add value to people’s lives and are a valuable contribution of the arts, although they can be intangible and difficult to define. The methodological limitations of the field notwithstanding, performing arts appear to offer promise as a therapeutic medium that may increase social contact, reduce social isolation, offer the uptake of new activities or the maintenance of pre-diagnosis activities and offer interventions that are relatively familiar to many people, including through exposure to popular performing arts competition shows on television – after all media, culture and society are closely interwoven [32].

### Previous literature reviews

Previous literature reviews on performing arts therapies for PD have focused largely on dance. Among recent efforts related to dance, systematic reviews of randomised controlled trials have shown a benefit on executive function [33] as well as motor symptoms and functional mobility, [34] while a systematic review of a broader range of studies [35] found that dance may have the potential to improve PD symptoms, particularly gait, global cognition and cognitive dual-tasking. There have been few published syntheses of the evidence relating to other performing arts modalities. One published systematic review has examined singing as a therapeutic modality in PD, conducted by our team, [36] and found evidence of benefit for speech, although all studies used a single-group repeated-measures design. A potential benefit of music therapy on gait in people with PD has been suggested by a systematic review, [37] although the music therapy interventions studied were not participatory. A non-systematic review [38] has however shown a benefit of instrument playing for PD, although this is limited by the non-systematic nature of the review. No review of theatrical interventions for PD has been published to our knowledge. There has been no systematic review assessing a full range of artistic modalities, precluding a truly comparative evaluation across modalities. This provides the rationale for conducting a new systematic review as presented in this manuscript.

### The present review

The current review sought to synthesise evidence relating to the clinical effectiveness of a broad range of performing arts modalities in PD in order to provide a comparative perspective not offered by existing reviews. Therefore, it was decided to narrow the focus to active participation interventions delivered in a group setting. Active participation excludes purely passive performing arts related activities such as listening to music, watching dancing or watching theatrical performances, and it is active participation that has been identified as having the power to alleviate behavioural and psychological symptoms as well as to benefit communication and relationships [39]. Furthermore, only group interventions were considered because these were considered most likely to address social challenges that have been shown to be important in PD. [27] The review was limited to clinical effectiveness on a set of five key outcome domains used in a previous review of singing in PD [36] – quality of life, speech, functional communication, motor function and cognitive status – as opposed to cost effectiveness or patient experience, for reasons of feasibility. The current review sought to answer the following questions:

1. What impact does group performing arts therapy, with active participation, have on the following outcomes for people with PD: quality of life, speech, functional communication, cognitive status or motor function?
2. Is there a difference in impact on these outcomes according to which performing arts modality is used?

It is only by considering all available active group-based performing arts interventions that a clear picture of the benefit of performing arts for PD can be ascertained. Offering this comparative perspective is the key step forward in knowledge offered by our manuscript.

## Methods

This review was conducted in accordance with PRISMA guidelines. The review was not pre-registered.

### Search strategy

Systematic searches were conducted in February 2020 using PsycINFO (Ovid), AMED (Ebsco), CINAHL (Ebsco), EMBASE (Ovid) and MEDLINE (Ovid). The published search strategy from a prior review of singing in PD [36] was expanded to encompass other performing arts modalities. The search strategy was developed in MEDLINE (Table 1) and translated for all other databases. Supplementary searches were conducted on Google Scholar and by screening reference lists of relevant retrieved articles. Forward and backward citation chasing were conducted on relevant reviews as well as studies identified for full-text screening. The results from each search were initially reviewed separately at the title and abstract stage. Those that appeared to meet the inclusion criteria were de-duplicated and combined. Initial full-text screening was performed based on language, article type and broad methodology. Then, detailed full-text screening was performed on all articles that remained in order to determine final inclusion. All screening procedures were performed independently by two persons.

**Table 1:** MEDLINE search strategy

“exp Parkinson disease/ AND singing.mp OR exp. Singing/ OR music.mp OR exp. Music/ OR music therapy.mp OR exp. Music therapy/ OR dance.mp OR dancing.mp OR exp. Dancing/ OR drama.mp OR exp. Drama/ OR theatre.mp OR theater.mp OR theatrical.mp OR performing art*.mp OR art OR arts or exp. Art/ OR art therapy.mp OR exp. Art therapy/”	

### Selection criteria

Studies were included in the review if 1) they looked at a performing arts intervention, 2) they assessed clinical effectiveness related to speech, functional communication, cognitive status, motor function or quality of life using any quantitative design, 3) the intervention offered an opportunity for active participation as opposed to passive appreciation, 4) they looked at group rather than individual interventions, 5) participants were people with a diagnosis of PD, and 6) they were published in a peer-reviewed journal in English (no date limit) or were published as an English-language conference abstract since January 2018.

### Data extraction and analysis

Data extracted from each study included study characteristics (Additional file 1: Appendix 1) – country, design, participants, inclusion criteria, and outcomes; intervention profile (Additional file 1: Appendix 2) – content, leader, location and duration of performing arts intervention; control profile (Additional file 1: Appendix 3) as well as results (Additional file 1: Appendix 4). All data extraction was performed independently by two persons using standardised forms, and any disagreements resolved by discussion. There was substantial methodological and clinical heterogeneity, including with regard to recruitment, settings, nature of the performing arts intervention, nature of the control arm and the assessment tools used for outcome measures. Therefore, thematic narrative synthesis was used as the primary analysis method. Additionally, random effects meta-analysis was performed for combinations of key scale outcomes (from UPDRS motor and TUG for motor function, MMSE, FAB and MoCA for cognitive function, and PDQ-39 total score for patient QoL) and interventions for which there were at least two studies using a common comparator. Review Manager version 5.3 software (Cochrane Collaboration) was used. In terms of performing arts interventions, singing, music therapy and theatrical interventions were assessed as unitary categories in this comparison. However, due to the wide variety of dance forms assessed by studies, we formed two categories 1) PD-specific dance forms and 2) tango or adapted tango, which were the two most common types of dance used in the studies. Outcomes were continuous and meta-analyses were conducted on mean difference between final follow-up and baseline (change score) where this information plus a measure of variability that could be converted to standard deviation was provided. If this information was not provided, meta-analyses were conducted on the scores at the final follow-up point. As the meta-analysis was a secondary analysis, there were no sensitivity or subgroup analyses, and risk of bias was considered at the level of the individual study, as outlined below. Forest plots are provided in Additional file 1: Appendix 7, Part C.

### Quality appraisal

All studies were quantitative, although they encompassed a variety of methods including randomised controlled trials, randomised trials with two intervention groups but no control group, non-randomised controlled trials, and single group repeated measures studies. In order to address the variation in designs, the Specialist Unit for Review Evidence (SURE) critical appraisal checklists (https://www.cardiff.ac.uk/specialist-unit-for-review-evidence/resources/critical-appraisal-checklists) were used. Previously used in evidence synthesis for performing arts interventions, [40] SURE is adapted from the Health Evidence Bulletins Wales checklist, the National Institute for Health and Care Excellence Public Health Methods Manual and the Critical Appraisal Skills Programme checklists. All versions of SURE ask similar questions, but are tailored to the specific study methodology. The SURE Experimental Studies Critical Appraisal Checklist was used for all trials, while the SURE Cohort Studies Critical Appraisal Checklist was used for longitudinal observational studies, such as single group repeated measures designs. SURE benefits from offering an in-depth appraisal rather than relying on a summary score, which may not provide an adequate assessment of limitations specific to the individual study [41]. Quality appraisal was conducted independently by two persons and was conducted at the study level. It was not possible to conduct quality appraisal for studies for which only conference abstracts were available due to insufficient information to conduct robust assessment. The results of the critique are shown in Additional file 1: Appendices 5 and 6. The results of the quality assessment were used to inform the interpretation and discussion of the findings.

## Results

**Fig. 1:**
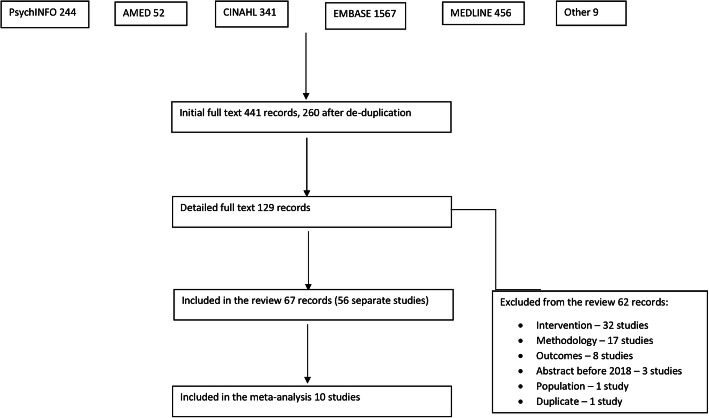
PRISMA flow chart for study selection

The study selection process resulted in the inclusion of 56 studies (reported in 67 separate publications) that met the inclusion criteria for the systematic review, of which 10 were able to provide data for the meta-analysis (see Fig. 1 for details of each stage in the process and reasons for exclusion). Studies came from 12 countries (Australia, Canada, Germany, Italy, Ireland, Japan, New Zealand, Norway, South Korea, Sweden, the UK, and the USA) and used a variety of quantitative designs. They were published from 1989 to 2020 and involved a total of 1531 participants with PD (sample size from one study not available); the number of participants ranged from 5 to 95 per study (median sample size 22). Studies covered four broad performing arts modalities: dance, music therapy, singing, and theatre. Music therapy was conceptualised as active interventions of a musical nature that did not solely involve singing. A full list of included studies is provided in Additional file 1: Appendix 8.

Theatre was the performing arts modality that was least studied with only two included studies [42, 43]. Both studies compared theatrical interventions led by professional performers to physiotherapy. Both studies were Italian, while one [43] randomised group allocation. In the other study [42], allocation to groups was determined by logistics rather than randomisation.

There were four studies assessing music therapy. Within this modality, each study was quite different in the music therapy intervention offered. Pohl et al. [44] assessed the Ronnie Gardiner Rhythm and Music Method (RGMM) comprising musical exercises to challenge cognition and sensorimotor control. Spina et al. [45] used an intervention that comprised musical exercises, singing and dancing, showing that interventions can draw on components of multiple performing arts. Pacchetti et al. [46] assessed the benefit of instrumental musical improvisation. Pantelyat et al. [47] considered a West African drum circle intervention and was the only music therapy study to not be randomised. Three studies compared music therapy to usual care, while the control in Pacchetti et al. [46] was a physiotherapy intervention.

There were 12 studies assessing singing interventions. Studies differed in the details of the intervention, but were all choral-based singing interventions. It was notable that the study by Tamplin et al. [48, 49] also included morning or afternoon tea for social interaction and conversation practice, which was offered to both intervention and control participants. Notably, in this study, the intervention was offered in weekly and monthly versions and the weekly and monthly singing groups differed in terms of having professional and amateur leaders respectively and whether the control group was a weekly activity such as painting, dancing or tai chi, or a monthly peer support group. Only two singing studies included a control group and the only randomised controlled trial was by Matthews et al., [50] in which the control group undertook a passive music appreciation activity.

As expected, given the greater focus on dance rather other performing art forms in previous reviews, dance was the performing arts therapeutic medium for which there was the largest body of evidence with a total of 38 studies (see Additional file 1: Appendix 1 for details of each study). Twenty-two of these dance studies included a control group: variously physiotherapy, [51] exercise, [52–58] education, [59–61] support groups, [62, 63] usual care, [55, 64–77] and a waiting list control [78]. Of these, there were 14 separate randomised controlled trials [51, 53–55, 57, 59, 61–70, 72, 75, 78, 79]. Across the 39 dance studies, a number of different dance styles were used. These could be broadly classified into PD-specific dance forms, such as the Dance for Parkinson’s Disease method as designed by the Mark Morris Dance Group and the Brooklyn Parkinson Group, [80] modern dance including improvisational dance forms, mixed-genre dance, Turo dance (based on Qi meridians), ballet, Irish set dancing, Ballu Sardu (a Sardinian folk dance) and tango (see Additional file 1: Appendix 2 for details on dance styles used in each study). Two separate forms of tango were used – traditional Argentine tango and adapted tango, the latter adapting steps for people with PD. Traditionally, in tango, the lead role is danced by the male. In adapted tango, typically all participants danced both lead and follow roles, while some studies of Argentine tango also adopted this practice. It was noted that in the single group repeated measures study by Koch et al. [81] on Argentine tango, there were three separate group workshops (with each participant attending one) and in the first workshop, the class was taught in English and translated into German, whereas the other two workshops had a different leader and were taught directly in German.

Methodological limitations were frequent and SURE analysis (Additional file 1: Appendices 5 and 6) highlights that common limitations included sampling, allocation methods and absence of control groups. The discussion reflects on these methodological issues and their implications.

### Narrative synthesis of outcomes for people with Parkinson’s disease

#### Quality of life

Twenty-two studies assessed the impact of dance interventions on quality of life (Additional file 1: Appendix 4), of which nine were randomised and eight had no control group. The most common dance interventions were PD-specific dance forms (9 studies) and tango or tango-based interventions (9 studies). Turo, Irish set dancing, American Ballroom and mixed-style partnered dance were all also studied. Across studies, the balance of the evidence supported a benefit of dance for quality of life, and this supported chiefly PD-specific and tango or tango-based dance forms, as these had been studied most. Only one study assessed American Ballroom [67–69] and found no evidence of significant benefit on quality of life. One study [59] found that following in tango offered greater quality of life benefit than leading. Four studies considered the impact of music therapy interventions on quality of life. Compared to usual care, Pantelyat et al. [47] found a beneficial effect of the drum circle, while Pohl et al. [44] found the same for RGRMM, as did Spina et al. [45] Compared to physiotherapy, Pacchetti et al. [46] found a beneficial effect of instrumental musical improvisation on health-related quality of life. Four studies assessed the impact of a singing intervention on quality of life [48–50, 82–85]. Of these, Matthews et al. [50] used a RCT design compared to a passive music appreciation activity, while Tamplin et al. [48, 49] used a non-randomised controlled trial design with various comparators, the limitations of which were discussed above. Studies differed as to what aspects of quality of life they assessed and how these were measured. General quality of life was assessed by three studies. Both Irons et al. [82, 83] and Matthews et al. [50] used the Parkinson’s Disease Questionnaire 39 Items (PDQ-39), [86], while Stegemöller et al. [84, 85] used the World Health Organization Quality of Life questionnaire (WHO-QOL) [87]. Voice-related quality of life (VRQoL) [88] was assessed by two studies [48, 49, 84, 85]. Additionally, Stegemöller et al. [84, 85] assessed swallow-related quality of life (SWAL-QOL), [89] although no significant effect was found on this outcome. Two studies considered the impact of theatrical interventions on quality of life. Mirabella et al. [42] and Modugno et al. [43] both found group theatrical interventions led by professional performers to be more effective than physiotherapy in improving overall health-related quality of life. Mirabella et al. [42] additionally found a greater benefit on emotional wellbeing.

#### Speech

Of the 12 studies considering singing interventions, 11 assessed speech outcomes. The body of evidence across studies (Additional file 1: Appendix 4) supports a benefit of group singing on speech, with only one study [90] finding no evidence of benefit. However, statistical significance was not always reached likely as a result of small sample sizes. Speech outcomes for which there may be benefit of group singing interventions include phonation, intelligibility and vocal intensity, although the precise patterning of speech features for which evidence of benefit was found differed between studies. Only Tanner et al. [91] reported clinical significance, and clinically significant improvements were found for intensity range in read speech and fundamental frequency variation, while the improvement in fundamental frequency in read speech was possibly clinically significant. Only two studies included a non-singing control group. In an RCT, Matthews et al. [50] compared a singing intervention to a passive music appreciation activity and found evidence of a significant benefit on phonatory measures. In a non-randomised controlled trial, Tamplin et al. [48, 49] compared weekly and monthly singing interventions to a weekly session of painting, dancing or tai chi or a monthly per support group, and found that singing significantly improved speech intensity but not phonation, while a greater benefit was found in the weekly group. It should be noted that the weekly singing intervention was delivered by a professional music therapist and the monthly singing intervention was delivered by recreational local musicians, which further complicates interpretation of the findings, since it is unclear whether it is weekly delivery or a professional teacher that drives the benefit. Moreover, both the intervention and control groups also attended a morning or afternoon tea alongside each session for socialising and conversational practice. No studies considered the impact of any other performing arts modalities besides singing on speech.

#### Functional communication

Two studies discussed the impact of singing interventions on functional communication. Shih et al. [90] found no significant change in functional communication after a group singing intervention of one 90-min session per week for 12 weeks. However, a study by Elefant et al. [92, 93] found that a group singing intervention of one 60-min session per week for 20 weeks significantly improved communicative facial expression and physical communication, although improvements in overall communication, plus functional and emotional subscales did not reach statistical significance. Neither study included a control group, which is a substantial limitation in terms of interpreting any observed benefit. No studies considered the impact of any other performing arts modalities besides singing on functional communication.

#### Cognitive status

Ten studies considered the impact of dance interventions on cognitive status (Additional file 1: Appendix 4), of which six were randomised and only one study [94] did not have a control group. The evidence sub-divides into PD-specific dance forms (5 studies), tango, either Argentine or adapted (4 studies) and Ballu Sardu (1 study). At least some evidence of benefit on cognition was found for all studies across both dance styles, except one study on PD-specific individually customised dance [62, 63]. One study [59] compared leading and following tango, and found that participants assigned to follow (this was not based on gender) improved significantly more in cognition than participants assigned to lead. Three studies considered the impact of music therapy interventions on cognitive status. Pohl et al. [44] found a benefit of the RGRMM on cognitive function, while Spina et al. [45] found the same with an active music therapy intervention comprising music, singing, and dancing. However, Pantelyat et al. [47] did not find evidence of a beneficial effect of a West African drum circle intervention on cognition. Among these three studies, all included a control group and in each case the control group was usual care. The studies by Pohl et al. [44] and Spina et al. [45] were both randomised. No studies considered the impact of singing interventions on cognitive status, although one study [82, 83] considered a cognitive quality of life subscale, which was considered a quality of life measure. One study [42] considered the impact of theatrical interventions on cognitive status and found no evidence of improvement in either the intervention or the physiotherapy control group. It used a non-randomised controlled trial design.

#### Motor function

Thirty-one studies considered the impact of dance interventions on motor function, of which 16 were randomised and eight lacked a control group (single group designs). The most common dance interventions were tango or tango-based dance (13 studies) and PD-specific dance forms (6 studies). Modern dance, improvisational dance, American Ballroom, mixed-genre or various partnered dance, ballet, turo, Irish set dancing, Ballu Sardu and dance/movement therapy were all also studied. Across studies, the balance of evidence supported a benefit of dancing for improving motor function, with the greatest volume of evidence being for tango and tango-related dance as well as PD-specific dance forms. One study [59] compared leading and following in tango and found that generally following was significantly motor effective than leading for improving motor function, although the opposite finding was found specifically for medication-related motor fluctuations. Only one study [95] considered clinical significance and found that the statistically significant benefit in motor function associated with improvisational dance fell slightly short of clinical significance. Four studies considered the impact of music therapy interventions on motor function. One study compared instrumental music improvisation to physiotherapy [46] and found that music therapy was more effective for improving motor function. RGRMM was found to be more effective than usual care for motor function, [44] while the evidence for a benefit of the drum circle on motor function was not conclusive [47] and no evidence of a benefit of the Spina et al. [45] music therapy intervention was found for motor function. One study considered the impact of a singing intervention on motor function. Using a design without a control group, but with high and low dosage intervention groups, which were allocated according to clinical and logistical factors rather than randomly, Stegemöller et al. [84, 85] found a benefit of a weekly group singing session for 8 weeks on the motor subscale of the Unified Parkinson’s Disease Rating Scale (UPDRS) [96]. Two studies considered the impact of theatrical interventions on motor function. Both compared to physiotherapy, one study [43] found evidence of a beneficial effect of theatre on motor function, while the other [42] did not.

#### Main methodological concerns

The main methodological concerns that were applicable to the body of evidence as a whole included small sample sizes, the absence of control groups in over half of the included studies (this was not an issue for the music therapy and theatrical studies, but was common in dance studies, and very common in singing studies), considerable variation in the frequency and duration of intervention delivery, a wide range of disciplinary backgrounds and levels of experience among session leaders, substantial heterogeneity of outcome measures, especially for cognition but also for motor function, as well as a focus on statistical rather than clinical significance. These issues are discussed in detail in the discussion section.

### Different performing arts modalities

No studies directly compared different performing arts modalities. Studies assessing each performing arts modality were conducted by different research teams, suggesting a tendency for scholars to work on a specific performing art modality rather than undertake multidisciplinary research across dance, music therapy, singing, and/or theatre. Not all of the outcomes of interest – speech, functional communication, cognitive status, motor function and quality of life – were assessed with regard to each performing art. In particular, speech and functional communication outcomes were only assessed in relation to singing interventions. Studies using singing interventions focused strongly on speech outcomes, with comparatively few considering a wider range of outcome domains. Furthermore, the volume of studies differed substantially between performing arts modalities. By far, the largest number of studies were conducted on dance interventions (39 studies) followed by singing interventions (12 studies). Comparably few studies assessed music therapy (not singing-only) interventions (4 studies) and theatrical interventions (2 studies). Among dance interventions, the greatest evidence was found for tango – either Argentine or adapted tango – as well as PD-specific tango forms, such as Dance for Parkinson’s Disease [80]. The body of evidence is currently insufficient to determine conclusively which performing arts modalities are most effective for which specific outcome domains due to the lack of comparative studies.

### Meta-analysis results

Following assessment of feasibility (Additional file 1: Appendix 7, Part A), six meta-analysis sets could be analysed, including a total of ten unique studies. Tabulated data for each comparison are shown in Additional file 1: Appendix 7, Part B, and forest plots in Additional file 1: Appendix 7, Part C.

The six feasible comparisons were: 1) UPDRS motor for tango-based dance vs exercise (3 studies), 2) UPDRS motor for tango-based dance vs usual care (2 studies), 3) UPDRS motor for theatre vs physiotherapy (2 studies), 4) TUG for PD-specific dance vs usual care (2 studies), 5) TUG for tango-based dance vs exercise (2 studies) and 6) PDQ-39 for PD-specific dance vs usual care (2 studies). Analyses were restricted to follow-up data except for comparison #6 for which the analysis could be conducted on change score data. The reason why change score based analyses could not be conducted for the other analysis sets was the unavailability of standard deviation data for the difference between baseline and follow-up scores for many studies, or a measure that could be converted into a standard deviation.

In analysis set one, meta-analysis did not show any evidence of a statistically significant difference in UPDRS motor scores at follow-up between participants undertaking tango-based dance and exercise (Z = 0.05, *p* = 0.96, Additional file 1: Appendix 7, Part C), although there were only three studies able to contribute to the meta-analysis and heterogeneity was a concern (I^2^ = 57%). In analysis set two, participants undertaking tango-based dance were statistically significantly superior on UPDRS motor at follow-up than participants undertaking exercise (Z = 2.87, *p* = 0.004, Additional file 1: Appendix 7, Part C), although there were only two studies and heterogeneity was a serious concern (I^2^ = 97%). In analysis set three, there was no statistically significant difference in UPDRS motor scores between participants undertaking theatrical interventions or physiotherapy (Z = 0.37, *p* = 0.71, Additional file 1: Appendix 7, Part C), although there were only two studies. In analysis set four, there was no statistically significant difference in TUG between participants undertaking PD-specific dance and usual care (Z = 0.98, *p* = 0.33, Additional file 1: Appendix 7, Part C), although there were only two studies and heterogeneity was a concern (I^2^ = 64%). In analysis set five, participants undertaking tango-based dance exhibited statistically significantly superior TUG scores at follow-up than participants undertaking exercise (Z = 11.25, *p* < 0.00001, Additional file 1: Appendix 7, Part C, although there were only two studies. In analysis set six, participants undertaking PD-specific dance experienced statistically significantly superior improvement in PDQ-39 from baseline to final follow-up than participants undertaking usual care (Z = 3.77, *p* = 0.0002, Additional file 1: Appendix 7, Part C).

## Discussion

### Summary

In relation to the first question of this review, the studies reviewed indicate that group performing arts interventions using active participation can impact positively on some of the symptoms experienced by people with PD, namely speech, cognition, motor function and quality of life. Potentially related to the dominance of motor features in the original conceptualisation of PD, [1] the greatest volume of evidence relates to motor symptoms. However, it is important to note this reflects that motor symptoms have been studied more often, not necessarily that the benefit of performing arts is greatest for this outcome domain. The evidence for functional communication outcomes remains too limited to draw any conclusions.

In relation to the second review question, it is difficult to assess which performing arts modalities are most effective for PD, either overall or in terms of improving particular outcome domains, since there were no studies comparing two or more different performing arts modalities. Few studies had two active interventions of a performing arts nature and where this was the case these were limited to either different doses of the same intervention or a comparison between two different dance forms. The greatest volume of evidence was for dance interventions. However, this shows that to date the greatest research interest into performing arts for PD has been in the modality of dance, not necessarily that it is more effective than other performing art modalities. It is difficult to compare performing arts modalities until all outcome domains have been assessed for all performing arts modalities. Nevertheless, the evidence indicates promise for all four performing arts modalities – dance, music therapy, singing and theatre – that they could benefit people with PD, at least in relation to certain symptoms. Within dance, the greatest promise appears to be with regard to tango and tango-related dance forms, as well as PD-specific dance. One study [59] showed a greater overall benefit of following than leading in tango dance for people with PD. This deserves further study, as it could have implications for gender equity in the clinical benefit offered by partnered dance forms for PD.

Considering the results of the meta-analysis, which must be interpreted with caution, of the six feasible meta-analysis comparisons, which did not encompass the full range of interventions, comparators and outcome measures in the systematic review, three comparisons showed statistically significant differences, all in favour of the performing arts arm. These were in analysis sets two, five and six. Participants undertaking tango-based dance were shown to have superior UPDRS motor and TUG scores than participants undertaking exercise, while participants undertaking PD-specific dance were shown to have superior PDQ-39 scores than participants undertaking usual care.

### Methodological considerations

The SURE checklists were used to appraise the quality of each included study. These indicated that methodological limitations were frequent and some applied to the majority of included studies.

#### Sample

There was a large variety in the locations of the studies included, both with regard to the country of study as well as the settings from which participants were recruited. This has implications in terms of variation in terms of routine clinical provision, but also in terms of societal familiarity with specific performing art forms, and cultural views both around performing arts and PD. Among performing arts, dance in particular is gendered and conceptions and norms relating to gender differ between cultures and countries. In some cultures, despite men typically performing the lead role in partnered dancing, there is a persisting cultural view that dance is not a masculine pursuit, [97] which is important to note given that men are at around 50% elevated risk of developing PD compared to women [98] and that therefore around 75% of people with PD are male. Male participants tended to be under-represented in the included studies (Additional file 1: Appendix 1), with many studies having a majority of female participants. This limits the generalisability of findings relating to performing arts interventions to male PD patients. Experiences and cultural meanings related to cognitive impairment are not universal and can be entwined with other contextual and cultural concepts such as expectations of ageing [99]. Specifically in PD, the psychological challenges of living with PD are closely linked to socio-cultural concepts including shame and stigma which differ between cultures and countries, [100] while healthcare practitioners’ perceptions of facial expressions in PD are influenced by culture and gender, with influence on perceptions of sociability and competence [101]. These various cultural factors relevant to both PD and the performing arts may exert a limiting influence on the extent to which study findings can be extrapolated beyond the contexts in which they were studied. The wide variability in sample sizes should also be taken into consideration, and this was a key limiting factor on the ability of many individual studies to draw more definitive conclusions. Moreover, there was generally a lack of detail about covariate structures in the statistical analysis and how this may address sampling challenges.

#### Outcome measures

A large variety of outcomes were studied with a variety of questionnaires and assessments used for the same construct. A wide variety of measures were used for motor function. However, there were certain assessments that featured frequently, such as the motor subscale of the UPDRS, either in its original [96] or revised version, [102] the Berg Balance Scale (BBS), [103] and the Timed Up and Go test (TUG), [104] which improves comparability between studies. Cognitive outcome measures used included the Mini Mental State Examination (MMSE), [105] the Montreal Cognitive Assessment (MoCA), [106] the Trail Making Test (TMT), [107] and the Frontal Assessment Battery (FAB), [108] although there was not a set of particular assessments that were used across the majority of studies, making comparability of cognitive results across studies difficult.

Studies assessing quality of life as a unitary concept mainly used the PDQ-39, [86] although other assessments such as the Oregon Health and Sciences University Quality of Life Scale [109] and the EuroQol-5D quality of life tool [110] were occasionally used. Some studies used other measures to assess more specific aspects of quality of life, such as Voice-Related Quality of Life (VRQoL) [88]. Studies assessing speech largely focused on common phonatory, articulatory, intensity, and intelligibility measures, although there were differences between studies in the exact ways these constructs were measured as well as the specific speech materials on which these measurements were made.

Only two studies assessed functional communication and both used the Voice Handicap Index (VHI), [111] as the outcome measure. While offering consistency, this measure does not solely measure communicative participation, [112] which is an important limitation in the assessment of functional communication since participation is the final common pathway for many aspects of function and disability [113]. Furthermore, any comparison of speech and communication measures across studies in an international systematic review faces intrinsic challenges related to the cross-linguistic and cross-cultural elements of this comparison since speech and communication are closely tied to the specific language and cultural context.

While outcome assessments were typically validated in the context in which they were developed, publications did not usually state whether and how the instruments had been validated in the country in which the study took place. It is important to not only to ensure that the translation is technically accurate, but that terms and concepts used have appropriate cultural connotations in order to avoid systematic differences in scoring at a population level compared to the country in which the instrument was originally developed. These issues are also important to consider in cases where the language is officially the same, but vocabulary and cultural concepts can differ substantially, such as using an instrument developed in the USA in a British context. Moreover, there is evidence, for example on quality of life questionnaires, [114] that there is a lack of methodological standardisation in the translation of outcome assessment tools. This may limit the comparability of studies conducted in different languages and countries. Additionally, one study offered questionnaires in both English and French, [57] which may restrict the internal validity of the outcome scores in this study.

#### Allocation

Many studies were not randomised and studies differed considerably in how they sampled participants, including in some cases from ongoing performing arts groups for PD in the community. The absence of randomisation was justified by some studies on ethical grounds and also for practical reasons, such as the availability of instructors in specific locations or during specific periods of time. Pragmatic and observational approaches to studying interventions may not be inferior [115]. While the absence of randomisation may increase the risk of confounding, especially if statistical adjustment methods such as propensity-score matching are not used, it is important for trials to correspond closely to clinical practice especially with regard to the patient profile, else the results may not able to be generalised to practice [116]. Some studies mention that assessors were blinded to group allocation. It is not possible to blind participants to the nature of the performing arts intervention that they are receiving. Some studies mention that they did not tell participants what the other group were receiving. This may reduce bias but the ethical aspects of this should also be considered.

#### Control groups

Less than half of studies included a control group and those that did used a wide variety of comparator interventions broadly categorised as physiotherapy, exercise, education, support groups, waiting list controls, music appreciation, other arts, and usual care. In the study by Tamplin et al., [48, 49] the control groups for the weekly and monthly singing interventions undertook fundamentally different activities from each other, making it difficult to assess the relative effectiveness of the two intervention frequencies. Usual care was the sole comparator in eleven studies, representing 41% of studies that included a control group. According to Smelt et al., [117] usual care as a control group exhibits substantial limitations and should be used with caution and described in as much detail as the intervention. Although descriptions of usual care were often adequate, it should be noted that usual care for PD is an elusive, vague and variant concept that may vary both between and within countries due to health system differences and clinician preferences, so a clearly defined control intervention may be preferable.

#### Intervention

Interventions were delivered by people from different disciplinary backgrounds and experience levels across studies within a given performing arts modality. Theatrical interventions were all given by professional performers, while music therapy interventions were all given (for those that provided this information) by either music therapists or music teachers. Greater variability in leaders was found in singing and dance interventions. Singing interventions were variously delivered by SLTs, music therapists, professional singing teachers, professional singers, recreational singers, and trained students or other facilitators, while the latter was variously delivered by professional dance instructors, professional dancers and recreational dancers, with varying levels of experience with PD. Additionally, there were studies in which all participants did not receive the intervention from the same instructor or from instructors with equivalent backgrounds and experience. The quality and focus of the intervention may have varied depending on the experience, training, disciplinary background and methodological or theoretical focus of the session leaders.

#### Limitations of the review

There were also certain limitations of the review process that should be taken into consideration. The review considered only English language literature. Secondly, while screening, study selection and quality assessment were conducted by two reviewers, only one researcher designed and conducted the searches – due to available expertise. Thirdly, only ten out of 56 included studies could contribute to meta-analysis, which also could not be conducted for all outcomes nor all combinations of interventions and comparators. This meant that meta-analysis could only form an additional analysis, and not the primary analysis of the manuscript, which was the narrative synthesis. Moreover, from six feasible analysis sets for meta-analysis, five were restricted to an analysis of follow-up data, due to non-availability of standard deviation data for the mean difference between baseline and follow-up scores for most studies for which such a change score was provided. Baseline differences between arms were generally not statistically significant in the included studies. However, any baseline differences, even small, could impact upon the reliability of meta-analyses in which solely follow-up data are compared.

### Clinical implications

When considering interventions for people with PD, this systematic review highlights the potential benefits of group performing arts interventions involving active participation, alongside appropriate pharmacotherapy. In the absence of clear comparative evidence regarding which performing arts modalities offer most benefit for particular outcomes, while there is evidence that all of dance, music therapy, singing and theatre may offer certain benefits, patient preference and logistics may be important considerations in terms of selecting which performing arts modality to select. Additionally, singing is the only modality so far that has been studied with regarding to speech outcomes, so this should be taken into consideration if patients’ particular concerns and difficulties relate to speech. It is important to note that suitable performing arts groups may already be available in the community, and that directing patients to these existing opportunities as a form of social prescribing, [118] may help participants’ sense of social integration as well as offering efficiencies in the health system organisation. The limitations of the evidence base as outlined above are also important to take into consideration.

### Research implications

Further research is needed with greater methodological rigour before firm conclusions can be drawn regarding which particular performing arts modalities offer the greatest benefits for people with PD and for which outcomes. No studies compared different performing arts modalities, for example a singing intervention with a theatrical intervention. Future research could compare these modalities through a combination of randomised controlled trials as well as robust large-scale real-life observational studies, [115] potentially drawing on existing PD performing arts classes that are offered in the community. With regard to trials, it is important that the design is well-matched to clinical need in order to ensure that they can provide useful information for clinicians as well as community service providers. While larger samples are important in future research, it is important that this is not at the expense of high levels of participant heterogeneity that could make the studies difficult to interpret for practical benefit. Moreover, given the underrepresentation of men in many studies, gender effects require further study, especially with regard to dance, due to the gendered nature of many dance forms. No studies reported whether there were differential gender effects of the intervention. One study [59] showed that following in tango was overall more beneficial for people with PD than leading. This could lead to partnered dancing being more beneficial for women than men, if dance follows traditional gender roles.

Moreover, there is a need to develop and agree standardised outcome sets to increase comparability of studies. The current review considered motor function, quality of life, cognitive function, speech and functional communication, although future studies and reviews could consider different outcome domains, including confidence and generalisation of skills. These would all benefit from consensus on which assessment instruments to use. It could be useful for all studies, irrespective of their primary focus, to include a quality of life instrument. The PDQ-39, which was the most commonly used quality of life instrument in included studies in the current review, has been considered as probably the most appropriate health related quality of life instrument in PD. [119] Greater focus on participation would enrich the evidence base for the current outcomes and others.

Participation is an important component of the World Health Organization’s [120] International Classification of Functioning, Disability, and Health (ICF), but has traditionally not been a great focus of research in PD, although there has been increased interest in recent years, which could be further expanded in the years to come. Only two studies in the current review assessed functional communication, despite this being an area of particular priority for people with PD. [24] This is an area of participation that in particular requires considerable further study. Future studies could use more modern outcome measures that are more focused on communicative participation such as the Communicative Participation Item Bank [121, 122] or the Communicative Effectiveness Survey [123, 124]. Indeed, communicative participation itself is a complex concept that is believed to be influenced by a range of PD-related factors including level of cognitive impairment, [125, 126] so would benefit from further study from a range of perspectives including in the context of performing arts interventions.

Moreover, future research and evidence syntheses should seek to address a wider range of outcomes relating to the potential benefit of performing arts interventions on outcomes in PD beyond clinical effectiveness. These outcomes could include adverse events, tolerability, participant experiences of the interventions, and the cost effectiveness of performing arts interventions for PD.

## Conclusion

Here we present the first systematic review to assess the potential benefit of all performing arts intervention modalities for people with PD. The results of this review highlight the potential positive use of group performing arts interventions – dance, music therapy, singing and theatre – with active participation for speech, motor function, cognition and quality of life in PD. However, methodological limitations, in particular the lack of studies comparing different performing arts modalities, make it difficult to conclude definitively which performing arts modalities offer the most benefit for people with PD, and whether different modalities are most beneficial for different outcome domains. The evidence regarding functional communication outcomes remains too limited to draw conclusions.

## Supplementary information

**Additional file 1:** **Appendix 1.** Study characteristics. **Appendix 2.** Intervention profile. **Appendix 3.** Control profile. **Appendix 4.** Results of included studies. **Appendix 5.** SURE critique checklist for experimental studies. **Appendix 6.** SURE critique checklist for cohort studies. **Appendix 7.** Meta-analysis. **Appendix 8.** Full list of included studies.

## Data Availability

This is a systematic review. All relevant information is provided in the manuscript and appendices.
