# Supplementary material for: A systematic review of active group-based dance, singing, music therapy and theatrical interventions for quality of life, functional communication, speech, motor function and cognitive status in people with Parkinson’s disease
Source: BMC Neurol. 2020 Oct 10;20:371. doi: 10.1186/s12883-020-01938-3 (PMC7547481; doi:10.1186/s12883-020-01938-3)
Supplement: Supplementary file 1 — Additional file 1: Appendix 1. Study characteristics. Appendix 2. Intervention profile. Appendix 3. Control profile. Appendix 4. Results of included studies. Appendix 5. SURE critique checklist for experimental studies. Appendix 6. SURE critique checklist for cohort studies. Appendix 7. Meta-analysis. Appendix 8. Full list of included studies. [file 12883_2020_1938_MOESM1_ESM.pdf]

APPENDICES

Appendix 1. **Study characteristics**

**First Author, year**	**Country**	**Design**	**Participants**	**Inclusion criteria**	**Outcomes**
<u>Dance</u>					
Allen, 2017; McKay, 2016	USA	Single group repeated measures study.	22 (7 male, mean age 65). Recruited at outreach events, day centres and/or care homes for senior citizens, and an outpatient movement disorders clinic.	IPD (Racette criteria). H&Y 1-4. Age≥35. No DBS, significant comorbidities or significant musculoskeletal impairment.	UPDRS-motor, dyskinesia, BBS, DGI, FABS, two-footed jump test, 6MWT, functional reach, single/dual TUG, gait analysis, ABC, FOG and response to perturbation.
Batson, 2010	USA	Single group repeated measures study.	11 (6 male, mean age 73). Convenience sample from a wellness centre at a teaching hospital.	IPD. Aged 50-85. Living independently in the community. No other neurological, cognitive or hearing problems.	TUG and FABS.
Batson, 2014	USA	Single group repeated measures study.	7 (2 male, mean age 67). Recruited from local area support groups and doctors.	NS.	FABS and TUG (including cognitive).

Bearss, 2017	Canada	Single group repeated measures study.	9 (5 male, mean age 68). Members of a new Dancing with Parkinson's Program at NBS.	NS.	BBS, TUG, Oregon QoL, Westheimer QoL and Heiberger QoL.
Blandy, 2015	Australia	Single group repeated measures study.	6 (3 male, mean age 64). Recruited from local and national PD support groups and movement disorder clinics.	IPD by neurologist. H&Y 1-3. Living in the community. Age 18-75. Medically safe to participate. MMSE ≥24	EQ-5D.
Clifford, 2017	UK	Single group repeated measures study (service evaluation).	7 (1 male, mean age 70). Recruited via local newspapers, local PD specialist services and a hospice.	PD.	PDQ-39.
De Natale, 2017	Italy	Non-randomised controlled trial.	16 (11 male, mean age 68). Recruited consecutively, but source NS.	PD (Gelb criteria). Responder to levodopa. MMSE >25.	UPDRS, BBS, DGI, TUG, 4SST, 6MWT, FAB, Stroop Test and TMT.
Duncan, 2014	USA	Randomised controlled trial.	10 (8 male, mean age 66). Recruited through a university movement disorders centre.	PD. Age >40. Receiving levodopa. No other serious medical condition.	MDS-UPDRS, Mini-BEST, gait analysis, TUG, 6MWT and FOG.
Duncan, 2012; Foster, 2013	USA	Randomised controlled trial.	52 (30 male, mean age 69). Recruited through a university movement disorders centre and	PD. H&Y 1-4. No other serious medical condition. Willing to miss doses for assessment.	MDS-UPDRS, Mini-BEST, gait analysis, FOG, 9HPT and ACS.

			advertisements in a local PD newsletter.		
Hackney, 2007 a,b	USA	Randomised controlled trial.	19 (12 male, mean age 71). Recruited from a university movement disorders centre. There were additionally age- and gender-matched 19 controls without PD.	PD (Racette crtieria). No other serious medical conditions. Vision corrected to 20/40 or better. Stand independently ≥30 minutes and walk independently ≥3m. MMSE >25.	ABC, mFES, functional reach, OLST, gait analysis, UPDRS, BBS and TUG.
Hackney, 2009 a,b,c	USA	Randomised controlled trial	61 (45 male, mean age 66). Community recruitment, including through a university movement disorders centre, local support groups and local community events.	IPD. Age ≥ 40. Stand ≥ 30 minutes and walk independently ≥3m. H&Y 1-3. No other neurological conditions. Benefit from levodopa. No serious uncorrected hearing or vision problems.	UPDRS-motor, BBS, TUG, 6MWT, FOG, gait analysis and PDQ-39.
Hackney, 2010	USA	Randomised trial.	39 (28 male, mean age 70). Community recruitment, including through a university movement disorders centre, local support groups and local community events.	IPD. H&Y 1-3. No other neurological conditions. Age ≥40. Stand ≥30 minutes and walk independently ≥3m. Benefit from levodopa.	BBS, tandem stance, one leg stance, TUG, 6MWT and gait analysis.

Hackney, 2018	USA	Randomised controlled trial.	83 (gender NS, mean age 70). Recruitment route NS.	PD.	6MWT, Corsi Blocks, MDS-UPDRS, ToL and PDQ-39.
Hashimoto, 2015	Japan	Randomised controlled trial.	46 (12 male, mean age 66). Recruitment through local PD associations.	PD. Living at home. Walk independently. Able to dance or exercise for an hour.	BBS, TUG, FAB, MRT and UPDRS.
Heiberger, 2011	Germany	Single group repeated measures study.	11 (5 male, mean age 71). Recruitment route NS.	Moderate-to-severe PD according to ICD-10 criteria.	UPDRS, TUG, Semitandem Test, Westheimer QoL and Oregon QoL.
Hulbert, 2017; Kunkel, 2017	UK	Randomised controlled trial.	51 (25 male, mean age 71). Recruited through PD support networks, consultants, regional research networks, newspaper advertisements and word of mouth.	PD by consultant. H&Y 1-3. Follow commands and remember instructions. No uncorrected visual or hearing impairments. No other neurological conditions.	Standing start 180 TT, BBS, Spinal mouse, ABC, 6MWT, TUG and PDQ-39.
Kalyani, 2019	Australia	Non-randomised controlled trial.	33 (13 male, mean age 65). Recruited through local PD support groups and websites, radio advertisements, an existing PD dance class and a university email list.	IPD (self-reported clinical diagnosis). Age 40-85. H&Y 1-3. ACE >82. No other serious medical conditions. Walk independently ≥3m.	NIH-COG, TMT, MDS-UPDRS ADL and PDQ-39.

Koch, 2016	Germany	Single group repeated measures study.	34 (8 male, mean age 61). Recruited through local PD support groups.	PD.	HSI and BSE.
Lee, 2018	South Korea	Randomised controlled trial.	32 (17 male, mean age 66). Recruited from a Korean Medicine hospital.	PD by neurosurgeon. Age 50-80. H&Y 1-3. No other neurological or cognitive conditions. No exercise therapy within 3 months.	UPDRS, PDQL and BBS.
McGill, 2019	UK	Non-randomised controlled trial.	32 (15 male, mean age 72). Intervention group recruited from an ongoing PD dance class. Control group recruited from local PD support groups and family members of intervention group.	PD. Age >55. No dementia (MMSE). Walk independently. No recent back surgery. No DBS.	Gait analysis and ABC.
McKee, 2013	USA	Non-randomised controlled trial.	33 (20 male, mean age 68 intervention and 74 controls). Recruited through flyers, referral, PD newsletters, PD support groups and websites.	IPD (Racette criteria). Aged over 50. H&Y 1-3. Benefit from PD. No other neurological conditions. Walk ≥3m.	MoCA, Reverse Corsi Blocks, BST, BBS, CPF and UPDRS motor.
McNeely, 2015	USA	Non-randomised trial.	22 (8 male, mean age 67). Recruited from a	PD by neurologist e.g. Racette criteria. Benefit from	MMSE, MDS-UPDRS motor, Mini-BEST, 6MWT,

			university movement disorders centre.	levodopa. Stand independently for 30 minutes. No evidence of dementia (MMSE ≥26). No other serious medical conditions. No DBS. No recent surgery or injuries affecting movement.	5TSS, 4SST, gait analysis, TUG, and PDQ-39.
McRae, 2018	USA	Single group repeated measures study.	61 (21 male, mean age 67). Recruited from well-established Dance for Parkinson's Disease classes.	People with PD.	8-item self-efficacy scale adapted from Lorig and a single item from SF-36.
Marchant, 2010	USA	Single group repeated measures study.	11 (7 male, mean age 71). Recruited from a university movement disorders centre and from the local area.	IPD (Racette criteria). Benefit from PD medication. Visual acuity of 20/40 with or without correction. Walk 3 m and stand 30 minutes. Normal somatosensory function in the feet. No other serious medical conditions.	UPDRS motor, BBS, TUG, 5TSS, 6MWT, gait analysis, FOG, ABC and PDQ-39.
Michels, 2018 a,b	USA	Randomised controlled trial.	13 (6 male, mean age 69). Recruited from a university movement disorders centre.	IPD by movement disorders expert. Stable medication regimen, no recent	H&Y, MDS-UPDRS, BBS, TUG, MoCA and PDQ-39.

				therapeutic dance or new PD treatments. MoCA ≥24.	
Patel, 2018	USA	Randomised controlled trial.	36 (25 male, mean age 68). Recruitment route NS.	PD. Experienced off time on the MDS-UPDRS medication-related motor fluctuations.	MDS-UPDRS-motor and medication-related motor fluctuations. Cognition and psychosocial function (tools NS).
Prewitt, 2017	USA	Single group repeated measures study.	6 (3 male, age range 62-87, mean NS). Recruited through routes including a local PD support group.	PD by physician. H&Y 1-3.	SCOPA-COG, S&E ADL, S&E ADL and GSE.
Rawson, 2019	USA	Non-randomised controlled trial.	74 in analysis (40 male, mean age 67)	IPD (Racette criteria). Age ≥30. Benefit from levodopa. H&Y 1-4. Walk 3m. No history of vestibular disease or dementia.	Gait analysis, Mini-BEST, MDS-UPDRS motor, H&Y, 6MWT and PDQ-39.
Rocha, 2018	Australia	Randomised trial.	21 (8 male, mean age 72). Recruited through PD support groups and medical clinics.	IPD. Modified H&Y 1-4. Stand ≥2 minutes. Walk independently ≥3m. Medical approval. MMSE ≥24. No comorbidities preventing exercise. No DBS.	Modified TUG, BBS, FGA, FOG, MDS-UPDRS and PDQ-39.

Romenets, 2015	Canada	Randomised controlled trial.	33 (19 male, mean age 63). Recruitment from a regional PD website and from a local movement disorders clinic.	IPD. H&Y 1-3. Stand ≥30 minutes. Walk ≥3 m. No dementia (MDS criteria), no severe hearing and vision problems, no change in PD medication in the past 3 months, no serious medical conditions or >3 falls in the past 12 months.	MDS-UPDRS motor and medication-related motor fluctuations, Mini-BEST, TUG, CCH, FOG, Purdue Pegboard, MoCA, and PDQ-39 – all in English or French..
Shanahan, 2017	Ireland	Randomised controlled trial.	41 in analysis (26 male, mean age 69). Recruited through clinicians and voluntary groups.	IPD. Modified H&Y 1-2.5. Walk 3m. No serious medical conditions or a hearing problem.	UPDRS motor, 6MWT, Mini-BEST and PDQ-39.
Shanahan, 2015	Ireland	Single group repeated measures study.	9 (7 male, mean age 66). Recruited through public talks.	IPD. Modified H&Y 1-2.5. Walk 3m. No serious medical conditions or a hearing problem.	UPDRS motor and PDQ-39.
Solla, 2019	Italy	Randomised controlled trial.	20 (13 male, mean age 67). Recruited from a hospital outpatient clinic.	PD (Gelb criteria). H&Y≤3. Walk independently. Stable medication regimen for 4 weeks. MMSE ≥24. No recent falls or history of other serious medical conditions.	UPDRS motor, 6MWT, BBS, TUG, 5TSS, back scratch, sit and reach, gait analysis and MoCA.

Ventura, 2016	USA	Non-randomised controlled trial.	15 (2 male – both in control group, mean age 71). Intervention group recruited from an existing PD dance program. Control group recruited from community-led PD support groups.	PD (self-reported diagnosis, not atypical). Aged 55-80. No previous dance experience. No other serious medical conditions. MMSE ≥ 25.	TUG, TGST, SBT, TEA, FES-I, VET, AF, AUT, WDS and PDQ-39.
Volpe, 2013	Italy	Randomised controlled trial.	24 (13 male, mean age 63). Recruited from a local PD association.	IPD by doctor. H&Y 0-2.5. No comorbidities that prevent dancing, mobility or safe exercise. No DBS.	UPDRS motor, TUG, BBS, modified FOG and PDQ-39.
Westbrook, 1989	USA	Non-randomised crossover study.	37 (group 1: 86% male, mean age 73, group 2: 40% male, mean age 70). Recruited from a local PD association.	PD.	Movement initiation.
Westheimer, 2015	USA	Single group repeated measures study.	12 (6 male, mean age 66). From a PD dance class, to which recruitment was through referral by other patients and from neurologists.	IPD by movement disorders specialist. H&Y 1-4. Age >30. Mobility with or without assistance. Ability to consent. Medical clearance.	H&Y, UPDRS motor, BBS and PDQ-39.
Zafar, 2017	USA	Non-randomised controlled trial.	35 (22 male, mean age 69). Recruited through flyers, referral, PD	IPD (Racette criteria). Benefit from PD medication.	IPA.

			newsletters, support groups and websites in the local area. There were additionally 74 older adults without PD.		
Music therapy					
Pacchetti, 2000	Italy	Randomised controlled trial	32 (23 male, mean age 63). Recruitment route NS.	IPD. Benefit from PD medication. H&Y 2-3. No cognitive impairment, visual or auditory deficits or other conditions affecting movement.	UPDRS and PDQ-39.
Pantelyat, 2016	USA	Non-randomised controlled trial	18 (7 male, mean age 71). Recruitment route from drum circle NS. Controls were recruited from an outpatient clinic.	IPD (Brain Bank criteria) and MDS-UPDRS motor. Having objective bradykinesia. Able to consent. Walk and stand, and to sit for 45-60 minutes.	MDS-UPDRS, TUG, PST, MoCA and PDQ-39.
Pohl, 2013	Sweden	Randomised controlled trial	18 (8 male, mean age 68). Recruited from a local PD support group.	Diagnosis of PD (not secondary or atypical). Stable treatment regimen. Able to squat. Walk ≥10m. Correctable auditory and visual	UPDRS, TUG, motion analysis based on the Posturo-Locomotion-Manual method, Cognitive Assessment Battery

				capability. No colour blindness, severe depression, or severe disability on UPDRS motor.	and PDQ39 Quality of Life tool.
Spina, 2016	Italy	Randomised controlled trial	25 (gender NS, mean age 68 intervention, 62 control, overall mean age and n per group NS). Recruitment route NS.	PD (Hughes criteria). Mild disability. On stable treatment. No dementia, depression, serious comorbidity or treatment that would affect cognition.	MDS-UPDRS, FAB, and PDQ-39.
Singing					
Azekawa, 2018	USA	Single group repeated measures study	5 (3 male, mean age 71). Recruited from a local PD support group.	PD. H&Y score 1-3. Age >50. No co-morbidity with other neurological or cognitive impairments. English as native language.	Sustained vowel phonation test, diadochokinesis test, Rainbow Passage reading – vocal function, voice quality, articulatory control ability and connected speech intelligibility.
Di Benedetto, 2009	Italy	Single group repeated measures study	20 (13 male, mean age 66). Consecutive referrals from a single rehabilitation centre.	PD (Brain Bank criteria). No history of substance abuse, psychiatric illness or head injury. MMSE ≥24.	Maximum phonation time, acoustic data from sustained vowel /ɑ/ production, quality of voice

					analysis prosody and fatigue ratings.
Elefant, 2012a,b	Norway	Single group repeated measures study	10 (7 male, mean age 64). Recruited from the neurology clinic of a single hospital.	PD. Stable levodopa response. H&Y score 2 or 3.	Fluency and acoustic data taken from a spoken passage and VHI. Facial expressions.
Evans, 2012	UK	Single group repeated measures study	17 (11 male, mean age 67). Recruited from the caseload of a county PD nurse specialist.	PD by doctor. Not requiring physical assistance during the session (unless a carer willing to attend).	FDA and PDQ-39.
Higgins, 2019	USA	Single group repeated measures study	10 (5 male, mean age 74). Recruited from a local PD support group.	Hypokinetic dysarthria secondary to IPD. Native speaker of Standard American English. Normal cognition. No depression or neurological comorbidity. No voice therapy within 12 months.	VSA and SIT.
Irons, 2020,2019	Australia, UK and South Korea	Single group repeated measures study	95 (43 male, mean age 70). Convenience sample recruited through PD support groups, social networks and radio advertising.	PD. No cognitive impairment.	PDQ-39.

Matthews, 2018	New Zealand	Randomised controlled trial	Sample size, characteristics and recruitment method NS.	NS.	Phonatory measures, PDQ-39 and cognitive function (tool NS).
Shih, 2012	USA	Single group repeated measures study	13 (11 male, mean age 66). Recruitment route NS.	PD (Brain Bank criteria). H&Y 1-5, VHI >8 and MMSE ≥24. No other voice therapy or involvement in other singing groups.	Acoustic data from Rainbow Passage and cookie theft picture description, VHI and VRQoL.
Stegemöller, 2017a,b	USA	Two group repeated measures study	27 (10 male, median age 69 in 'low dosage' group and 64 in 'high dosage' group). Recruitment method NS.	IPD. Non-smoking. Stable medication regimen. No other serious medical conditions. MMSE ≥24. Beck ≥18.	Voice measures, UPDRS, SWAL-QoL, VRQoL and WHO-QoL.
Tamplin, 2019,2018	Australia	Non-randomised controlled trial	75 (46 male, mean age 74). Recruited from local PD support groups.	PD by neurologist (MDS criteria). MMSE ≥17. No memory problems, severe language difficulties or hearing impairment.	Voice, speech and VRQoL.
Tanner, 2016	Canada	Single group repeated measures study	28 (14 male, mean age 65). Recruited from community groups.	PD by neurologist, H&Y≤3 and sufficient skills to participate.	Acoustic data including from spontaneous monologue and reading Grandfather Passage.

Yinger, 2016	USA	Single group repeated measures study	10 (7 male, mean age 70). Convenience sample.	PD. Not reporting medication change during study.	Acoustic data from Rainbow Passage.
<u>Theatre</u>					
Mirabella, 2017	Italy	Non-randomised controlled trial	24 (10 male, mean age 60). Recruited from hospital clinics and local PD associations.	IPD, H&Y 2-3, stable medication regimen. MMSE >24. Absence of severe sensory deficits or motor disability.	UPDRS, GFQ, S&E, PDQ-39 and a neuropsychological battery.
Modugno, 2010	Italy	Randomised controlled trial	20 (10 male, mean age 63). Recruited from hospital outpatient clinics.	IPD, H&Y 2-4, on a stable medication regimen. No severe visual or auditory deficits or movement dysfunctions.	UPDRS, S&E and PDQ-39.

ABC = Activities-specific Balance Confidence scale (Powell & Myers, 1995), ACE = Addenbrooke's Cognitive Examination (Mathuranath, Nestor, Berrios, Rakowicz, & Hodges, 2000), ACS = Activity Card Sort (Baum & Edwards, 2008), ADL = Activities of Daily Living, AF = action fluency, AUT = Alternative Uses Test (Guilford, 1967), Beck = Beck Depression Inventory (Beck, 1972), BBS = Berg Balance Scale (Berg, Wood-Dauphinée, Williams, & Gayton, 1989), Brain Bank criteria = UK Parkinson's Disease Society Brain Bank criteria (Gibb & Lees, 1988), BSE = Body Self-Efficacy, BST = Brooks Spatial Test (Brooks, 1967), CCH = Canadian Community Health Survey Falls Questionnaire – Health Ageing adapted (Statistics Canada, 2008), CPF = Composite Physical Function Index (Rikli & Jones, 2001), DGI = Dynamic Gait Index (Shumway-Cook & Woollacott, 1995), DBS = deep brain stimulation, EQ-5D = EuroQol 5 Dimensions Quality of Life scale (EuroQol Group, 1990), FAB = Frontal Assessment Battery (Dubois, Slachevsky, Litvan, & Pillon, 2000), FABS = Fullerton Advanced Balance Scale (Rose, Lucchese, & Wiersma, 2006), FDA = Frenchay Dysarthria Assessment (Enderby, 1983), FES-I = Falls Efficacy Scale – International (Yardley et al, 2005), FGA = Functional Gait Assessment (Wrisley, Marchetti, Kuharsky, & Whitney, 2004), FOG = Freezing of Gait questionnaire (Giladi et al, 2000), Gelb criteria = Gelb, Oliver, & Gilman, 1999, GFQ = Gait and Falls Questionnaire, GSE = General Self-Efficacy, Heiberger QoL = Heiberger et al (2011) Quality of Life Scale, HSI = Heidelberg State Inventory (Koch et al, 2016), Hughes criteria = Hughes, Daniel, Kilford, & Lees, 1992, H&Y = Hoehn and Yahr staging (Hoehn & Yahr, 1967); IPA = Impact on Participation and Autonomy questionnaire (Sibley et al, 2006), IPD = idiopathic Parkinson's disease, Lorig = Lorig, Chastain, Ung, Shoor, & Holman, 1989, MDS = Movement Disorders Society, MDS-UPDRS = Movement Disorders Society sponsored revision of the Unified Parkinson's Disease Rating Scale (Goetz et al, 2008), mFES = Modified Falls Efficacy Scale (Edwards & Lockett, 2008), Mini-BEST – Mini Balance Evaluation Systems Test (Franchignoni, Horak, Godi, Nardone, & Giordano, 2010), MMSE = Mini Mental State Examination (Folstein, Folstein, & McHugh, 1975), MoCA = Montreal Cognitive Assessment (Nasreddine et al, 2005), MRT = Mental rotation test, NBS = National Ballet School, NIH-COG = National Institutes of Health Toolbox Cognition Battery (Weintraub et al, 2013), NS = not stated, OLST = One leg stand test, Oregon QoL = Oregon Health and Sciences University Quality of Life scale (Bearss, McDonald, Bar, & DeSouza, 2017), PD = Parkinson's disease, PDQ-39 = Parkinson's Disease Questionnaire – 39 Items (Peto, Jenkinson, Fitzpatrick, & Greenhall, 1995) , PDQL = Parkinson's Disease Quality of Life questionnaire (de Boer, Wijker, Speelman, & de Haes, 1996), PST = Postural sway test, Racette criteria = Racette, Rundle, Parsian, & Perlmutter (1999), SBT = Standing balance test, S&E = Schwab & England (Schwab & England,1969), SCOPA-COG = SCales for Outcomes in PArkinson's disease-COGnition (Marinus et al, 2003), SF-36 = 36-Item Short Form Health Survey (Saris-Baglama et al, 2007), SIT = Sentence Intelligibility Test (Yorkston, Beukelman, & Tice, 1996), SLT = Speech and Language Therapy/ist, SWAL-QoL = Swallow-Related Quality of Life (McHorney et al, 2002), TEA = Test of Everyday Attention (Robertson, Ward, Ridgeway, & Nimmo-Smith, 1994), TGST = Timed gait speed test, TMT = Trail Making Test (Army Individual Test Battery, 1944), ToL = Tower of London (Shallice, 1982), TUG = Timed Up and Go (Podsiadlo & Richardson, 1991), UPDRS = Unified Parkinson's Disease Rating Scale (Fahn, Elton, & UPDRS Program Members, 1987), VET = Visual elevator test, VHI = Voice Handicap Index (Jacobson et al, 1997), VRQoL = Voice-

Related Quality of Life (Hogikyan & Sethuraman, 1999), VSA = Vowel space area, WDS = Wechsler Digit Span (Wechsler, 1997), Westheimer QoL = Westheimer (2008) Quality of Life Scale, WHO-QoL = World Health Organization Quality of Life scale (WHOQOL Group, 1994), 4SST = Four square step test, 5TSS = Five times sit to stand, 6MWT = Six minute walking test, 9HPT = Nine hole peg test, 180 TT = 180 degrees turn test.

Appendix 2. **Intervention profile**

**First author, year**	**Content**	**Leader**	**Location**	**Duration**
Dance				
Allen, 2017; McKay, 2016	Adapted tango.	A professional dance instructor.	A large multipurpose university room.	15 group sessions of 90 minutes over 3 weeks.
Batson, 2010	Modern dance.	A dance teacher experienced in teaching the elderly.	A large multipurpose room in a wellness centre.	3 group sessions of 85 minutes per week for 3 weeks.
Batson, 2014	Improvisational dance: seated, at the ballet Barre and ambulating.	Two dance instructors trained in improvisational dance.	An accessible dance studio.	3 group sessions of 1 hour per week for 7 weeks.
Bearss, 2017	Dance for Parkinson's Disease.	Two NBS faculty members trained in Dance for Parkinson's Disease.	NBS Canada.	1 group session of 75 minutes per week for 12 weeks.
Blandy, 2015	Argentine tango.	A professional dance instructor.	A dance studio.	2 group sessions of 1 hour per week for 4 weeks.
Clifford, 2017	Dance for Parkinson's programme, including improvisational and creative dance tasks.	A dance artist, who is an experienced Dance for Parkinson's practitioner.	A hospice.	1 group session of 90 minutes per fortnight for 12 weeks.
De Natale, 2017	Argentine tango.	A professional dance instructor.	NS	2 group sessions of 60 minutes per week for 10 weeks.
Duncan, 2014	Argentine tango.	Two volunteers who are experienced Argentine tango dancers.	A community-based location.	2 group sessions of 1 hour per week for 2 years.
Duncan, 2012; Foster, 2013	Argentine tango. Participants danced both lead and follow roles.	A tango instructor.	A community-based location.	2 group sessions of 1 hour per week for 1 year.

Hackney, 2007 a,b	Argentine tango. All participants danced both lead and follow roles.	A professional dance instructor.	NS.	2 group sessions of 1 hour per week for a total of 20 sessions over 13 weeks.
Hackney, 2009 a,b,c	Argentine tango.<br>American ballroom: waltz/foxtrot.<br>All participants danced both lead and follow roles.	An experienced professional ballroom dance instructor who was also a certified personal trainer.	NS.	2 group sessions of 1 hour per week for a total of 20 sessions over 13 weeks.
Hackney, 2010	Based on Argentine tango. Partner and Non-partner positions. All participants performed lead and follow roles.	An experienced professional ballroom instructor who was also a certified personal trainer.	NS.	2 group sessions of 1 hour per week for 10 weeks.
Hackney, 2018	Adapted tango. Participants assigned to lead or follow exclusively.	NS.	NS.	20 group sessions of 90 minutes over 13 weeks. Frequency NS.
Hashimoto, 2015	PD-specific dance: alone, in pairs and in groups.	NS.	NS.	1 group session of 60 minutes per week for 12 weeks.
Heiberger, 2011	Dance for Parkinson's disease.	A professional dancer.	A ballet studio.	1 group session of 75 minutes per week for 8 months.
Hulbert, 2017; Kunkel, 2017	Partnered dance based on basic ballroom and Latin steps. Dance steps differed by gender following tradition.	Two experienced ballroom and Latin dance teachers.	NS.	2 group sessions of 1 hour per week for 10 weeks.
Kalyani, 2019	Dance for Parkinson's Disease.	Dance for Parkinson's Disease trained instructors.	NS.	2 group sessions of 1 hour per week for 12 weeks.
Koch, 2016	Argentine tango.	Workshop 1: a dance movement therapist and	NS.	1 group session of 90 minutes in total. Three

		tango teacher from Argentina (session translated from English)<br>Workshops 2 and 3: a dance movement therapy advanced student and tango teacher from Germany.		separate workshops were run attended by different participants.
Lee, 2018	Turo: a dance form based on the Qi meridian system.	A Turo instructor.	A Korean Medicine hospital.	2 group sessions of 60 minutes per week for 8 weeks.
McGill, 2019	Ballet.	Dance artists in the hosting ballet company's outreach department.	A ballet dance studio.	1 group session of between 75 and 90 minutes per week in term time (3 terms per year lasting 10-12 weeks) for 1 year.
McKee, 2013	Adapted tango.	Dance instructors without clinical experience.	Retirement communities.	20 group sessions of 90 minutes over 12 weeks.
McNeely, 2015	Dance for Parkinson's Disease.<br>Tango. All participants danced both lead and follow roles.	Dance for Parkinson's Disease: an undergraduate student with pre-professional ballet and modern dance experience.<br>Tango: two graduate students who were experienced tango dancers.	A community-based group setting on a university campus.	2 group sessions of 1 hour per week for 12 weeks.
McRae, 2018	Dance for Parkinson's disease.	NS – due to recruitment for assessment of participants from various established dance classes.	NS – due to recruitment for assessment of participants from various established dance classes.	Participants were in established dance classes and the pattern and frequency differed.

Marchant, 2010	Short duration, high dose contact improvisation dance workshop.	A professional improvisational dance instructor.	NS.	10 group sessions of 90 minutes over a 2 week period.
Michels, 2018 a,b	Dance therapy for Parkinson's disease. A customised session catered to the individual.	A certified dance therapist with experience teaching people with PD.	A movement studio at a sports institute.	1 group session of 1 hour per week for 10 weeks.
Patel, 2018	Adapted tango.	NS.	NS.	30 hours of group sessions over 12 weeks.
Prewitt, 2017	Let's Dance!	Two academic physiotherapists (recreational dancers).	A university physiotherapy laboratory skills classroom.	2 group sessions of 1 hour per week for 8 weeks.
Rawson, 2019	Argentine tango.	Tango dance instructors.	University facilities.	2 group sessions of 1 hour per week for 12 weeks.
Rocha, 2018	Argentine tango. Mixed-genre: comprised tap dancing, creative dance and Irish dancing.	Two experienced dance teachers.	A dance venue with a wooden floor, barre and mirrors.	1 group session of 1 hour for 8 weeks with a concurrent home dance programme.
Romenets, 2015	Argentine tango.	Two professional tango instructors without expertise in PD.	A dance studio.	2 group sessions of 1 hour per week for 12 weeks.
Shanahan, 2017	Irish set dancing.	Irish set dancing teachers who were either also clinicians or experienced in teaching clinical populations.	A community venue.	1 group session of 90 minutes per week for 10 weeks. Parallel home programme.
Shanahan, 2015	Irish set dancing.	A set dancing teacher who was also a chartered physiotherapist.	A community hall.	1 group session of 90 minutes per week for 8 weeks. Parallel home programme.

Solla, 2019	Ballu Sardu (a Sardinian folk dance).	A Sardinian folk dance teacher.	NS.	2 group sessions of 90 minutes per week for 12 weeks.
Ventura, 2016	Dance for Parkinson's Disease.	Two trained Dance for Parkinson's Disease instructors.	NS.	1 group session of 75 minutes per week for 10 weeks – for some participants these were not consecutive.
Volpe, 2013	Irish set dancing.	Two set dancing teachers.	A dance studio.	1 group session of 90 minutes per week for 6 months. Supplementary home programme.
Westbrook, 1989	Dance/movement therapy: development of a movement theme facilitated by the therapists.	The authors – a search suggests they are psychologists.	The halls of two suburban churches.	Group sessions of 60 minutes for 6 weeks. It is not clearly stated whether it is one session per week.
Westheimer, 2015	Dance for Parkinson's Disease.	Dance teachers who developed the Dance for Parkinson's Disease method	Mark Morris Dance Center.	2 group sessions of 75 minutes per week for 8 weeks.
Zafar, 2017	Adapted tango.	Tango instructors.	Retirement communities.	20 group sessions of 90 minutes within 12 weeks. Classes were twice weekly.
<u>Music therapy</u>				
Pacchetti, 2000	Instrumental music improvisation: piano, organ, percussion instruments and a hi-fi system.	A music therapist.	NS.	1 group session of about 2 hours per week for 13 weeks.

Pantelyat, 2016	West African drum circle class.	Local African drumming instructors.	Dance studio at a university movement disorders centre.	2 group sessions of 45 minutes to 1 hour per week for 6 weeks.
Pohl, 2013	Ronnie Gardiner Rhythm and Music Method.	A certified Ronnie Gardiner Rhythm and Music Method practitioner.	NS.	2 group sessions of 1 hour per week for 6 weeks.
Spina, 2016	Music, singing and dancing.	NS.	NS.	1 group session of 90 minutes per week for 24 weeks.
<u>Singing</u>				
Azekawa, 2018	Well-known songs.	A trained graduate student supervised by music therapists.	NS.	1 group session of 50 minutes per week for 6 weeks.
Di Benedetto, 2009	Choral singing using modified popular and liturgical chants accompanied on the piano.	An SLT who is an expert choral singer.	A hospital chapel.	1 group session of 2 hours per week for 13 weeks. Prior to this, there was a series of vocal exercise sessions.
Elefant, 2012 a,b	Songs from the Beatles as well as Norwegian folk songs accompanied on the guitar.	A music therapist.	A familiar room in the hospital’s rehabilitation centre.	1 group session of 1 hour per week for 20 weeks.
Evans, 2012	Call and response singing to well-known tunes then singing songs.	A professional singing teacher with a personal interest in PD.	NS.	1 group session of 2 hours per fortnight for 2 years.
Higgins, 2019	Singing.	A trained vocal performer with a master’s degree in Music.	NS.	1 group session of 90 minutes per week for 11 weeks. Participation in 9 sessions was required for continuation.

Irons, 2020, 2019	Sing to Beat Parkinson's.	Trained facilitators.	NS.	1 group session of 1 hour per week for 6 months.
Matthews, 2018	Singing, voice and respiration exercise.	NS.	NS.	1 group session per week for 9 weeks – session duration not stated.
Shih, 2012	Singing popular songs.	An SLT who was also a singing instructor.	NS.	1 group session of 90 minutes per week for 12 weeks.
Stegemöller, 2017 a,b	Group singing of familiar songs.	Music therapists.	NS.	1 group session per week for 8 weeks – session duration NS. There was also a complementary home programme.
Tamplin, 2019, 2018	Singing popular and traditional songs and rounds.	Weekly: a music therapist. Monthly: community musicians and volunteers.	NS.	1 group session of 2 hours weekly or monthly for 3 months.
Tanner, 2016	Vocal exercises followed by melody and song singing, accompanied by a pianist.	An SLT who is also a classically trained singer.	NS.	2 group sessions of 90 minutes per week for 6 weeks
Yinger, 2016	Singing exercises using songs selected by the participant and accompanied on the guitar or piano.	A music therapist.	NS.	2 group sessions of 50 minutes per week for 6 weeks.
<u>Theatre</u>				
Mirabella, 2017	Movement, vocal and theatre training: the latter comprising vocal technique, improvisation and experimentation, and dramaturgy.	Professional performers (an actor and either a singer or a dancer).	NS.	1 group session of 3 hours per week for 15 months.

Modugno, 2010	Vocal warm-up, preparation of the scene and staging.	Professional actors.	NS.	2 or 4 (alternating monthly) group sessions of 6 hours per month for 3 years.

Adapted tango = steps from Argentine tango adapted to suit people with PD with all participants dancing both lead and follow roles regardless of gender unless stated, Argentine tango = danced in traditional gender roles unless stated, Dance for Parkinson's Disease = a model developed by Mark Morris Dance Center and Brooklyn Parkinson Group including modern dance, choreography and partner dancing (Westheimer, 2008), Let's Dance! = a university-led PD-specific dance class with dances from a large repertoire of partner, group and line dances: the bachata, ballroom waltz, Texas two-step, polka, shim-sham, swing, Cajun waltz, foxtrot, samba, tango, square dance, electric slide, meringue, samba, and barangara, NBS = National Ballet School, NS = Not stated, PD = Parkinson's disease, Ronnie Gardiner Rhythm and Music Method = musical exercises that challenge cognition and sensorimotor control, Sing to Beat Parkinson's = a programme developed by the investigators which after warm-ups features singing participants' preferred songs, SLT = Speech and Language Therapy/ist.

Appendix 3. **Control profile**

**First author, year**	**Synopsis of control arm**
Dance	
De Natale, 2017	Traditional rehabilitation: 2 group sessions of 1 hour per week for 10 weeks. Static and dynamic balance exercises, and gait training.
Duncan, 2014	Usual care.
Duncan, 2012; Foster, 2013	Usual care.
Hackney, 2007 a,b	Traditional exercise: 2 group sessions of 1 hour per week – completing 20 sessions within 13 weeks. Structured traditional strength/flexibility chair exercise class.
Hackney, 2009 a,b,c	Usual care.
Hackney, 2018	Wellness education: 20 group sessions of 90 minutes over 13 weeks.
Hashimoto, 2015	PD exercise: 1 group session of 60 minutes for 12 weeks.<br>Usual care.
Hulbert, 2017; Kunkel, 2017	Usual care.
Kalyani, 2019	Usual care.
Lee, 2018	Waiting list control.
McGill, 2019	Usual care – asked not to take dance classes during the study.
McKee, 2013	Education: 20 group sessions of 90 minutes over 12 weeks. Seminars on diverse health-related topics to encourage interaction and socialising.
Michels, 2018 a,b	Support group: 1 group session of 60 minutes per week for 10 weeks. Traditional talking therapy support group facilitated by a professional counsellor.
Patel, 2018	Education: 30 hours of group sessions over 12 weeks. Socially supportive classes addressing health and wellness topics relevant to older adults with PD.
Rawson, 2019	Treadmill: 2 group sessions of 60 minutes per week for 12 weeks.<br>Stretching: 2 group sessions of 60 minutes per week for 12 weeks.

Romenets, 2015	Self-directed exercise: a wait list control group that was additionally given a booklet about exercise in PD produced by the Parkinson Society of Canada.
Shanahan, 2017	Usual care.
Solla, 2019	Usual care.
Ventura, 2016	Usual care.
Volpe, 2013	Routine physiotherapy: individual sessions of 80 minutes covering movement, stretching, strength training, balance training, postural re-education and gait training. Participants had an average of 21 sessions over 6 months.
Westbrook, 1989	Exercise group: Structured routine of exercises including rowing movements, windmill movements of the arms, and neck exercises. The exercise classes lasted for 6 weeks. The session duration and frequency are not stated.
Zafar, 2017	Usual care.
<u>Music therapy</u>	
Pacchetti, 2000	Physiotherapy: weekly group sessions of 90 minutes for 13 weeks.
Pantelyat, 2016	Usual care.
Pohl, 2013	Usual care.
Spina, 2016	Usual care.
<u>Singing</u>	
Matthews, 2018	Music appreciation: watching and discussing music videos in a group once a week for 9 weeks – session duration not stated.
Tamplin, 2019,2018	Weekly control: a weekly session of painting, dancing or tai chi. Monthly control: a monthly peer support group.
<u>Theatre</u>	
Mirabella, 2017	Physiotherapy: group sessions of 1.5 hours 2 days a week for 15 months.
Modugno, 2010	Physiotherapy: individual sessions of 2-3 hours 3 days a week for 3 years.

PD = Parkinson's disease.

Appendix 4. **Results of included studies**

**First author, year**	**Results**
Dance	
Allen, 2017; McKay, 2016	There was evidence that adapted tango significantly improved motor function, including through physiological assessment.
Batson, 2010	There was evidence that modern dance significantly improved balance, while the difference in TUG fell short of statistical significance.
Batson, 2014	There was evidence that improvisational dance significantly improved balance (although this fell slightly short of clinical significance), while the difference in TUG fell short of statistical significance.
Bearss, 2017	There was evidence that Dance for Parkinson's Disease significantly improved motor function, although this was not found for quality of life.
Blandy, 2015	There was evidence that Argentine tango improved quality of life, although statistical significance was not reached.
Clifford, 2017	There was evidence that a Dance for Parkinson's programme improved quality of life, although statistical significance was not reached.
De Natale, 2017	There was evidence that Argentine tango was significantly more effective for motor and cognitive functions than traditional rehabilitation exercises.
Duncan, 2014	There was evidence that Argentine tango was significantly more effective for motor function than usual care, and that these gains were sustained for two years while the usual control group deteriorated.
Duncan, 2012; Foster, 2013	There was evidence that Argentine tango was significantly more effective for motor function than usual care.
Hackney, 2007 a,b	There was evidence that Argentine tango was significantly more effective for motor function than traditional exercises.
Hackney, 2009 a,b,c	There was evidence that tango but not American Ballroom dancing significantly improved health-related quality of life. Both tango and

	American Ballroom dancing significantly improved motor function versus no intervention, but the effect was stronger for tango.
Hackney, 2010	There was evidence that tango significantly improved motor function (gait and balance) and that this did not differ significantly between partnered and non-partnered conditions.
Hackney, 2018	There was evidence that following rather than leading tango was significantly more beneficial overall for motor function, cognition and quality of life, although leading was more effective for motor fluctuations.
Hashimoto, 2015	There was evidence that PD-specific dance was significantly more effective than PD exercise or usual care in improving motor and cognitive symptoms.
Heiberger, 2011	There was evidence that Dance for Parkinson's Disease significantly improved motor function (with the strongest effect being on rigidity). A significant impact on quality of life was also found, particularly relating to recreation, socialising and social impact.
Hulbert, 2017; Kunkel, 2017	There was evidence that partnered dance significantly improved motor function (though not all on measures), although this effect was not found for quality of life.
Kalyani, 2019	There was evidence that Dance for Parkinson's disease significantly improved cognition and quality of life compared to usual care.
Koch, 2016	There was evidence that tango significantly improved quality of life, assessed by measures of well-being and body self-efficacy.
Lee, 2018	There was evidence that Turo dance significantly improved quality of life compared to a waiting list control. There was some evidence for a significant benefit on motor function, being found on UPDRS-motor but not a balance assessment.
McGill, 2019	There was no evidence that ballet significantly improved motor function, considering gait and balance confidence, compared to usual care.
McKee, 2013	There was evidence that adapted tango was significantly more effective than education in improving motor function and cognition.

McNeely, 2015	There was evidence that tango significantly improved motor function, while motor symptoms deteriorated in the Dance for Parkinson's Disease group. Gait analysis variables did not however change significantly in either group. Cognitive status was assessed but results post-intervention were not reported. There was no significant effect of either dance intervention on quality of life.
McRae, 2018	There was evidence that Dance for Parkinson's Disease improves quality of life, including self-efficacy. Mediation analysis showed that one way in which higher levels of functional mobility influence overall quality of life is through enhanced self-efficacy.
Marchant, 2010	There was evidence that contact improvisation dance improved motor function. Quality of life was measured but results were not reported.
Michels, 2018 a,b	There was evidence that a customised PD dance intervention improved motor function and quality of life, but this was not shown for cognition. The study was not designed to assess whether differences versus a support group control were significant.
Patel, 2018	There was evidence that adapted tango was significantly more effective than an educational intervention for motor function, cognitive function and quality of life.
Prewitt, 2017	There was evidence that a Let's Dance! programme significantly improved quality of life (self-efficacy and ADL measures) and some evidence of a significant benefit for cognitive function, although this was not found for all measures.
Rawson, 2019	There was no evidence that tango significantly improved motor function or quality of life.
Rocha, 2018	There was evidence that Argentine tango significantly improved mobility, balance and motor disability, while mixed-genre dance significantly improved freezing of gait. There was a trend to improved quality of life in Argentine tango participants (but not mixed-genre dance participants), although this did not reach statistical significance.

Romenets, 2015	There was some evidence that Argentine tango was significantly more effective than self-directed exercise for motor function, although this effect was not found for the primary outcome measure UPDRS-motor. There was no evidence of a significant effect on quality of life. Tango participants displayed greater improvement in cognition, although statistical significance was not reached.
Shanahan, 2017	There was no evidence that Irish set dancing significantly improved motor function. However, Irish set dancing improved quality of life more than usual care, although statistical significance was not reached.
Shanahan, 2015	There was evidence that Irish set dancing significantly improved quality of life. There was also an improvement in motor function, although statistical significance was not reached.
Solla, 2019	Ballu Sardu offered significantly greater benefits for motor and cognitive function than usual care.
Ventura, 2016	There was evidence that Dance for Parkinson’s Disease was more than usual care for motor function and cognition, although effects were not consistent across measures. Large effect sizes were found for measures of cognitive switching, attention, gait speed and falls efficacy. Evidence of a significant benefit on quality of life was also found.
Volpe, 2013	There was evidence that Irish set dancing was significantly more effectively than physiotherapy exercise in improving motor function. For quality of life, both groups improved, but there was no significant difference.
Westbrook, 1989	There was evidence that dance/movement therapy was significantly more effective than exercise in improving movement initiation.
Westheimer, 2015	There was evidence that Dance for Parkinson’s Disease significantly improved motor function, but this effect was not found for quality of life.
Zafar, 2017	There was evidence that adapted tango was significantly more effective than usual care in improving quality of life outcomes related

	to participation and autonomy, including social life, autonomy indoors and family role subscales.
Music therapy	
Pacchetti, 2000	There was evidence that instrument-based music therapy was significantly more effective than physiotherapy for motor function and quality of life.
Pantelyat, 2016	There was evidence that the drum circle intervention improved quality of life significantly more than usual care. There was some evidence that the drum circle intervention improved motor function significant more than usual care. There was no evidence for a significant beneficial effect of the drum circle intervention on cognition.
Pohl, 2013	There was evidence that the Ronnie Gardiner Rhythm and Music Method significantly improved motor function, cognition and quality of life, which did not improve significantly in the usual care control group.
Spina, 2016	There was evidence that active music therapy significantly improved cognition and quality of life significantly more than usual care, although this effect was not found for motor function.
Singing	
Azekawa, 2018	There was evidence that singing improved phonatory, intelligibility and fluency, although statistical significance was not consistently reached.
Di Benedetto, 2009	There was some evidence that singing significantly improved phonation, although this was not found for all phonatory measures.
Elefant, 2012a,b	There was some evidence that singing significantly improved functional communication (including facial expression), although this was not found for all measures. There was no evidence of a significant improvement in spoken fluency, intensity or phonatory measures.

Evans, 2012	There was evidence that singing significantly improved phonation and intensity, although this evidence was not found for intelligibility or quality of life.
Higgins, 2019	There was evidence that singing significantly improved intelligibility and articulation (vowel space area).
Irons, 2020,2019	There was evidence that singing improved quality of life, with statistical significance being reached for emotional well-being, cognition and communication quality of life subscales. An effect on the social support subscale was found, but it was moderated by country with the effect being found only in South Korean and not Australian or British participants.
Matthews, 2018	There was evidence that singing significantly improved phonatory, cognition and quality of life measures.
Shih, 2012	There was no evidence that singing significantly improved phonatory, intensity or functional communication measures.
Stegemöller, 2017a,b	There was evidence that singing significantly improved motor function, quality of life, and voice-related quality of life. There was some evidence that singing significantly improved phonatory measures, although this was not found for all measures. There was no evidence of a significant benefit on swallow-related quality of life.
Tamplin, 2019,2018	There was evidence that singing significantly improved speech intensity and voice-related quality of life, but not phonation. Weekly participants improved more than monthly participants.
Tanner, 2016	There was some evidence that singing significantly improved intensity and phonation, although this was not found for all measures. Clinically significant improvements were found for intensity range in read speech and fundamental frequency variation, while the improvement in fundamental frequency in read speech was possibly clinically significant.
Yinger, 2016	There was some evidence that singing significantly improved intensity, but this was not found for all measures. There was no evidence that singing significantly improved phonation.

Theatre	
Mirabella, 2017	There was evidence that theatre was significantly more effective than physiotherapy in improving quality of life (including emotional wellbeing). Neither the theatre nor the physiotherapy group improved significantly in terms of motor function or cognition.
Modugno, 2010	There was evidence that theatre significantly improved motor function and quality of life, whereas physiotherapy did not.

ADL = Activities of daily living, TUG = Timed Up and Go, UPDRS = Universal Parkinson's Disease Rating Scale.

Appendix 5. **SURE critique checklist for experimental studies**

a. **Dance studies**

**SURE critical appraisal checklist questions**	De Natale, 2017	Duncan, 2014	Duncan, 2012; Foster, 2013	Hackney, 2007a,b	Hackney, 2009a,b,c	Hackney, 2010
Does the study address a clearly focused question/hypothesis?	Yes.	Yes.	Yes.	Yes.	Yes.	Yes.
Was the population randomised? If yes, were appropriate methods used?	No.	Yes, NS.	Yes, online.	Yes, NS.	Yes, hat.	Yes, hat.
Was allocation to intervention or comparator groups concealed?	No.	No.	No.	No	Yes – in so far as participants didn't know study purpose.	Yes – in so far as participants didn't know study purpose.
Were participants/ investigators blinded to group allocation? If no, was assessment of outcomes blinded?	NS, yes.	No, yes.	No, yes.	No, yes.	No, yes.	Participants, yes.
Were interventions (and comparisons) well described and appropriate?	Yes.	Unclear.	Yes.	Yes.	Yes.	Yes.
Was ethical approval sought and received?	Yes.	Unclear, was in line with policies.	Yes.	NS.	Yes.	Yes.

Was a trial protocol published?	NS.	NS.	Yes.	NS.	NS.	NS.
Were the groups similar at the start of the trial?	Yes.	Yes.	Yes.	Unclear.	Yes.	Yes.
Was the sample size sufficient?	Unclear.	Unclear.	Yes.	Unclear.	Unclear.	Yes.
Were participants properly accounted for?	Unclear.	Unclear.	Unclear.	Unclear.	Unclear.	Yes.
Are the statistical methods well described?	No.	Unclear.	Unclear.	No.	Unclear.	Unclear.
Results appropriate and clear?	Yes.	Yes.	Yes.	Unclear.	Yes.	Yes.
Is there any sponsorship/conflict of interest stated?	Charity, no conflict.	Charity, no conflict.	Charity and academic, no conflict.	Charity, no conflict.	Charity and state, no conflict.	Charity and state, no conflict.
Did the authors identify any limitations?	Yes.	Yes.	Yes.	No.	Yes.	Yes.
Are the conclusions the same in the abstract and full text?	No, abstract stronger.	No, full text stronger.	No, full text stronger.	Unclear.	Unclear.	Unclear.

**SURE critical appraisal checklist questions**	Hashimoto, 2015	Hulbert, 2017; Kunkel, 2017	Kalyani, 2019	Lee, 2018	McGill, 2019	McKee, 2013
Does the study address a clearly focused question/hypothesis?	Yes.	Yes.	Yes.	Yes.	Yes.	Yes.
Was the population randomised? If yes, were appropriate methods used?	Yes, coin.	Yes, phone.	No.	Yes, online.	No.	No.
Was allocation to intervention or comparator groups concealed?	No.	No.	No.	No.	No.	No.
Were participants/ investigators blinded to group allocation? If no, was assessment of outcomes blinded?	No, yes.	No, no.	No, no.	No, yes.	No, no.	No, partly.
Were interventions (and comparisons) well described and appropriate?	Yes.	Yes.	Yes.	Yes.	Yes.	Yes.
Was ethical approval sought and received?	Yes.	Yes.	Yes.	Yes.	Yes.	Yes.
Was a trial protocol published?	NS.	NS.	Yes.	NS.	NS.	NS.

Were the groups similar at the start of the trial?	Yes.	Yes.	Yes.	Yes.	Unclear.	Yes.
Was the sample size sufficient?	Yes.	Yes.	Yes.	Unclear.	Unclear.	No.
Were participants properly accounted for?	Unclear.	No.	Unclear.	Yes.	Unclear.	Unclear.
Are the statistical methods well described?	Unclear.	Unclear.	Unclear.	Unclear.	Unclear.	Yes.
Results appropriate and clear?	Yes.	Yes.	Yes.	Yes.	Yes.	Yes.
Is there any sponsorship/conflict of interest stated?	No funding.	Academic, no conflict.	Academic, dance teaching conflicts declared.	State, no conflict.	Academic, state and commercial, no conflict declared.	State, no conflict.
Did the authors identify any limitations?	Yes.	Yes.	Yes.	Yes.	Yes.	Yes.
Are the conclusions the same in the abstract and full text?	Unclear.	Yes.	Yes.	No, full text stronger.	Unclear.	Unclear.

**SURE critical appraisal checklist questions**	McNeely, 2015	Michels, 2018a,b	Rawson, 2019	Rocha, 2018	Romenets, 2015	Shanahan, 2017
Does the study address a clearly focused question/hypothesis?	Yes.	Yes.	Yes.	Yes.	Yes.	Yes.
Was the population randomised? If yes, were appropriate methods used?	No.	Yes, online.	No.	Yes, online.	Yes, online.	Yes, envelopes.
Was allocation to intervention or comparator groups concealed?	No.	No.	No.	Yes, in so far as they were blind to aims.	Unclear.	No.
Were participants/ investigators blinded to group allocation? If no, was assessment of outcomes blinded?	NS.	No, yes.	No, yes.	Dance teachers yes, yes.	Unclear, no.	No, yes.
Were interventions (and comparisons) well described and appropriate?	Yes.	Yes.	Yes.	Yes.	Yes.	Yes.

Was ethical approval sought and received?	Yes.	Yes.	Yes.	Yes.	Yes.	Yes.
Was a trial protocol published?	NS.	NS.	NS.	NS.	Yes.	Yes.
Were the groups similar at the start of the trial?	Yes.	No.	Yes.	Yes.	Yes.	Yes.
Was the sample size sufficient?	Unclear.	No.	Yes.	Unclear.	Unclear.	Yes.
Were participants properly accounted for?	Unclear.	Unclear.	Unclear.	Yes.	Yes.	No.
Are the statistical methods well described?	Yes.	Unclear.	Yes.	Yes.	Yes.	Unclear.
Results appropriate and clear?	Yes.	Yes.	Yes.	Yes.	Yes.	Yes.
Is there any sponsorship/conflict of interest stated?	State, no conflict.	No funding. One author declares commercial conflicts.	Charity, state and academic, no conflict.	Academic and commercial, no conflict declared.	Charity and state, the authors declare no conflict of interest for this study.	NS.
Did the authors identify any limitations?	Yes.	Yes.	No.	Yes.	Yes.	Yes.
Are the conclusions the same in the abstract and full text?	Unclear.	Unclear.	Unclear.	Yes.	No, abstract stronger.	Yes.

**SURE critical appraisal checklist questions**	Solla, 2019	Ventura, 2016	Volpe, 2017	Westbrook, 1989	Zafar, 2017
Does the study address a clearly focused question/hypothesis?	Yes.	Yes.	Yes.	Unclear.	Yes.
Was the population randomised? If yes, were appropriate methods used?	Yes, online.	No.	Yes, online.	No.	No.
Was allocation to intervention or comparator groups concealed?	No.	No.	No.	No – cross-over.	No.
Were participants/ investigators blinded to group allocation? If no, was assessment of outcomes blinded?	No, yes.	No, yes.	No, yes.	No – cross-over.	NS.
Were interventions (and comparisons) well described and appropriate?	Yes.	Yes.	Yes.	Yes.	Yes.
Was ethical approval sought and received?	Yes.	Yes.	Yes.	NS.	Yes.
Was a trial protocol published?	NS.	NS.	Yes.	NS.	NS.

Were the groups similar at the start of the trial?	Yes.	Unclear.	Yes.	Yes.	Unclear.
Was the sample size sufficient?	Yes.	No.	Unclear.	Unclear.	Unclear.
Were participants properly accounted for?	Unclear.	Unclear.	Unclear.	Unclear.	Unclear.
Are the statistical methods well described?	Unclear.	Unclear.	No.	Unclear.	No.
Results appropriate and clear?	Yes.	No.	Yes.	Unclear.	Yes.
Is there any sponsorship/conflict of interest stated?	Charity, no conflict.	Charity, state and academic, no conflict.	Not stated, no conflict declared.	Charity, no conflict	Charity, state and academic, no conflict.
Did the authors identify any limitations?	Yes.	Yes.	Yes.	Yes.	Yes.
Are the conclusions the same in the abstract and full text?	Unclear.	Unclear.	No, abstract stronger.	Unclear.	No.

25 trials in total – quality assessment could not be undertaken for Hackney, 2018 and Patel, 2018 as they comprised only an abstract.

b. Music therapy studies

**SURE critical appraisal checklist questions**	Pacchetti, 2000	Pantelyat, 2016	Pohl, 2013	Spina, 2016
Does the study address a clearly focused question/hypothesis?	Unclear.	Unclear.	Yes.	Unclear.
Was the population randomised? If yes, were appropriate methods used?	Yes, online.	No.	Yes, online.	Yes, NS.
Was allocation to intervention or comparator groups concealed?	No.	No.	No.	No.
Were participants/ investigators blinded to group allocation? If no, was assessment of outcomes blinded?	No, yes.	No, yes.	No, yes.	No, yes.
Were interventions (and comparisons) well described and appropriate?	Yes.	Yes.	Yes.	No.
Was ethical approval sought and received?	NS.	Yes.	Yes.	NS.
Was a trial protocol published?	NS.	NS.	NS.	NS.

Were the groups similar at the start of the trial?	Unclear.	Yes.	Yes.	Yes.
Was the sample size sufficient?	Unclear.	Unclear.	Yes.	Unclear.
Were participants properly accounted for?	Unclear.	Unclear.	Unclear.	Unclear.
Are the statistical methods well described?	Yes.	No.	No.	No.
Results appropriate and clear?	Yes.	Yes.	No.	No.
Is there any sponsorship/conflict of interest stated?	State, no conflict.	Charity, academic and state, no conflict.	Academic and state, no conflict.	NS, no conflict declared.
Did the authors identify any limitations?	No.	Yes.	Yes.	No.
Are the conclusions the same in the abstract and full text?	Unclear.	No, abstract stronger.	No, abstract stronger.	No abstract.

4 trials in total.

c. **Singing studies**

**SURE critical appraisal checklist questions**	Tamplin, 2018, 2019
Does the study address a clearly focused question/hypothesis?	Yes.
Was the population randomised? If yes, were appropriate methods used?	No.
Was allocation to intervention or comparator groups concealed?	No.
Were participants/ investigators blinded to group allocation? If no, was assessment of outcomes blinded?	No, partly.
Were interventions (and comparisons) well described and appropriate?	No – weekly and monthly controls were different activities. Weekly and monthly interventions had leaders with different backgrounds.
Was ethical approval sought and received?	Yes.
Was a trial protocol published?	Yes.

Were the groups similar at the start of the trial?	Unclear.
Was the sample size sufficient?	Yes.
Were participants properly accounted for?	Unclear.
Are the statistical methods well described?	No.
Results appropriate and clear?	Yes.
Is there any sponsorship/conflict of interest stated?	Charity and academic, no conflict
Did the authors identify any limitations?	Yes.
Are the conclusions the same in the abstract and full text?	Yes.

2 trials in total – quality assessment could not be undertaken for Matthews, 2018 as it comprised only an abstract.

d. **Theatre studies**

**SURE critical appraisal checklist questions**	Mirabella, 2017	Modugno, 2010
Does the study address a clearly focused question/hypothesis?	Unclear.	Unclear.
Was the population randomised? If yes, were appropriate methods used?	No.	Yes, online.
Was allocation to intervention or comparator groups concealed?	Yes, in so far as participants were not told that the other group involved.	Yes, in so far as participants were not told that the other group involved.
Were participants/ investigators blinded to group allocation? If no, was assessment of outcomes blinded?	No, yes.	No, yes.
Were interventions (and comparisons) well described and appropriate?	Yes.	Yes.
Was ethical approval sought and received?	Yes.	Yes.
Was a trial protocol published?	NS.	NS.

Were the groups similar at the start of the trial?	Yes.	Yes.
Was the sample size sufficient?	Unclear.	Unclear.
Were participants properly accounted for?	Unclear.	Unclear.
Are the statistical methods well described?	Yes.	Unclear.
Results appropriate and clear?	Yes.	Unclear.
Is there any sponsorship/conflict of interest stated?	Charity, no conflict.	State, no conflict.
Did the authors identify any limitations?	Yes.	Yes.
Are the conclusions the same in the abstract and full text?	Unclear.	Unclear.

2 trials in total. NS = not stated. ‘Online’ randomisation refers to randomisation techniques using computerised algorithms.

Appendix 6. **SURE critique checklist for cohort studies**

a. Dance studies

**SURE critical appraisal checklist questions**	Allen, 2017; McKay, 2016	Batson, 2010	Batson, 2014	Bearss, 2017	Blandy, 2015
Is the study design clearly stated?	Yes.	Yes.	Yes.	Unclear.	Yes.
Does the study address a clearly focused question?	Yes.	Unclear.	Yes.	Unclear.	Yes.
Are the setting, locations and relevant dates provided?	Partly.	Partly.	Partly.	Partly.	Partly.
Were participants fairly selected?	No.	No.	Unclear.	Unclear.	Unclear.
Are the measures of exposures and outcomes appropriate?	Yes.	Yes.	Yes.	Yes.	Yes.
Was bias considered?	Yes.	Unclear.	Unclear.	Unclear.	Yes.
Is there a description of how the study size was arrived at?	Yes.	No.	No.	No.	Unclear.

Are the statistical methods well described?	Yes.	No.	No.	No.	No.
Is information provided on participant flow?	Yes.	Partly.	No.	Partly.	Yes.
Are the results well described?	Yes.	No.	No.	Partly.	Yes.
Is there any sponsorship/ conflict of interest reported?	Charity, state and academic, no conflict.	Academic, no conflict.	NS, no conflict declared.	Charity and academic, no conflict.	No funding.
Did the authors identify any limitations?	Yes.	No.	Yes.	No.	Yes.

**SURE critical appraisal checklist questions**	Clifford, 2017	Heiberger, 2011	Koch, 2016	McRae, 2018	Marchant, 2010
Is the study design clearly stated?	No.	Unclear.	Yes.	Unclear.	Yes.
Does the study address a clearly focused question?	No.	Unclear.	Yes.	Unclear.	Yes.
Are the setting, locations and relevant dates provided?	Partly.	Partly.	Yes.	Partly.	Partly.
Were participants fairly selected?	Unclear.	Unclear.	Unclear.	Unclear.	Unclear.
Are the measures of exposures and outcomes appropriate?	Yes.	Yes.	Unclear – dance classes attended by some participants were translated.	Yes.	Yes.
Was bias considered?	Unclear.	Unclear.	Yes.	Yes.	Unclear.
Is there a description of how the study size was arrived at?	No.	No.	Partly.	No.	No.
Are the statistical methods well described?	No.	No.	No.	Yes.	Yes.

Is information provided on participant flow?	Partly.	No.	No.	No.	No.
Are the results well described?	Partly.	Partly.	Yes.	Partly.	Partly.
Is there any sponsorship/ conflict of interest reported?	‘External funding’, no conflict declared.	NS, no conflict declared.	No funding.	NS, no conflict declared.	Charity and academic, no conflict.
Did the authors identify any limitations?	Partly.	Partly.	Yes.	Yes.	Yes.

**SURE critical appraisal checklist questions**	Prewitt, 2017	Shanahan, 2015	Westheimer, 2015
Is the study design clearly stated?	Unclear.	No.	Yes.
Does the study address a clearly focused question?	Yes.	Yes.	Unclear.
Are the setting, locations and relevant dates provided?	Partly.	Partly.	Partly.
Were participants fairly selected?	Unclear.	Unclear.	Unclear.
Are the measures of exposures and outcomes appropriate?	Yes.	Yes.	Yes.
Was bias considered?	Unclear.	Unclear.	Unclear.
Is there a description of how the study size was arrived at?	No.	No.	Partly.

Are the statistical methods well described?	Yes.	No.	Unclear.
Is information provided on participant flow?	No.	No.	No.
Are the results well described?	Yes.	Partly.	Partly.
Is there any sponsorship/ conflict of interest reported?	NS, no conflict declared.	NS, no conflict declared.	No funding.
Did the authors identify any limitations?	Yes.	Yes.	Yes.

13 studies in total.

b. Music therapy studies

No cohort studies.

c. Singing studies

**SURE critical appraisal checklist questions**	Azekawa, 2018	Di Benedetto, 2009	Elefant, 2012a,b	Evans, 2012	Higgins, 2019
Is the study design clearly stated?	Yes.	Yes.	Yes.	Yes.	Partly.
Does the study address a clearly focused question?	Partly.	Yes.	Yes.	Yes.	Yes.
Are the setting, locations and relevant dates provided?	Partly.	Partly.	Partly.	Yes, except the exact venue.	Partly.
Were participants fairly selected?	Unclear.	Yes, probably.	Unclear.	Unclear.	Unclear.
Are the measures of exposures and outcomes appropriate?	Yes.	Yes.	Yes.	Yes.	Yes.
Was bias considered?	Yes.	Unclear.	Unclear.	Unclear.	Unclear.
Is there a description of how the study size was arrived at?	No.	No.	No.	No.	No.

Are the statistical methods well described?	No.	No.	No.	No.	No.
Is information provided on participant flow?	Partly.	No.	No.	No.	No.
Are the results well described?	Yes.	No.	Partly.	Partly.	Partly.
Is there any sponsorship/ conflict of interest reported?	NS.	Charity, no conflict.	NS.	NS.	No funding.
Did the authors identify any limitations?	Yes.	Partly.	Yes.	Yes.	Yes.

**SURE critical appraisal checklist questions**	Irons 2019, 2020	Shih, 2012	Stegemöller, 2017a,b	Tanner, 2016	Yinger, 2016
Is the study design clearly stated?	Yes.	Yes.	Yes.	Partly.	Yes.
Does the study address a clearly focused question?	Yes.	Unclear.	Yes.	Yes.	Yes.
Are the setting, locations and relevant dates provided?	Partly.	Partly.	Partly.	Partly.	Partly.
Were participants fairly selected?	No.	Unclear.	Unclear.	Unclear.	No.
Are the measures of exposures and outcomes appropriate?	Yes.	Yes.	Yes.	Yes.	Yes.
Was bias considered?	Yes.	No.	Unclear.	Yes.	Unclear.
Is there a description of how the study size was arrived at?	No.	Yes, although design was changed.	No.	Partly.	No.
Are the statistical methods well described?	Unclear.	No.	Unclear.	Unclear.	No.

Is information provided on participant flow?	Partly.	Partly.	No.	No.	No.
Are the results well described?	Yes.	Yes.	Partly.	Partly.	Partly.
Is there any sponsorship/ conflict of interest reported?	Charity and academic, no conflict.	Charity, state, academic and commercial, no comment on conflict.	Charity, no conflict.	Charity, no conflict.	NS.
Did the authors identify any limitations?	Yes.	Yes.	Yes.	Yes.	Yes.

10 studies in total.

d. Theatre studies

No cohort studies. NS = Not stated. Note: In Evans et al (2012), the acknowledgements provide the name of the partner of one of the participants and say that this partner provided ‘organizational support’ to the study. No further information on this was available.

Appendix 7. **Meta-analysis**

Part A. **Assessment of feasibility**

The standardised scales considered for use in meta-analysis were UPDRS motor (either original version or MDS revision) and TUG for motor function, MMSE, FAB and MoCA for cognitive function and PDQ-39 total score for QoL.

In order to conduct a meta-analysis, a minimum of two studies was required for a given outcome measure for a given combination of intervention and comparator. Singing, music therapy, and theatrical interventions were each treated as unitary categories in this comparison. Dance was sub-divided into 1) PD-specific dance forms and 2) tango-based dance.

**Outcome**	**Intervention**	**Comparator**	**N studies**	**References**	**Notes**
UPDRS	PD-specific dance	Education	0		
		Exercise	1	Hashimoto et al [56]	
		Music appreciation	0		
		Painting, dancing or tai chi	0		
		Physiotherapy	0		
		Support group	1	Michels et al [63-64]	
		Usual care	2	Hashimoto et al [56]<br>Kalyani et al [72]	Hashimoto [56] excluded as only total UPDRS reported – meta-analysis not feasible
		Waiting list	0		
	Tango-based dance	Education	3	Hackney et al [60]<br>McKee and Hackney [61]<br>Patel et al [62]	Hackney et al [60] and Patel et al [62] excluded as no mean + SD values reported – meta-analysis not feasible
		Exercise	4	De Natale et al [53]<br>Hackney et al [54-55]<br>Rawson et al [57]<br>Romenets et al [58]	Rawson et al [57] excluded as no mean +SD values reported at follow-up

		Music appreciation	0		
		Painting, dancing or tai chi	0		
		Physiotherapy	0		
		Support group	0		
		Usual care	3	Duncan and Earhart [65] Duncan and Earhart [66] /Foster et al [67] Hackney and Earhart [68-70]	Duncan and Earhart [65] excluded as no mean +SD values reported at follow-up.
		Waiting list	0		
	Singing	Education	0		
		Exercise	0		
		Music appreciation	0		
		Painting, dancing or tai chi	0		
		Physiotherapy	0		
		Support group	0		
		Usual care	0		
		Waiting list	0		
	Music therapy	Education	0		
		Exercise	0		
		Music appreciation	0		
		Painting, dancing or tai chi	0		
		Physiotherapy	1	Pacchetti et al [47]	
		Support group	0		
		Usual care	3	Pantelyat et al [48] Pohl et al [45] Spina et al [46]	Pantelyat et al [48] and Pohl et al [45] excluded as no mean + SD values reported at follow-up –

					meta-analysis not feasible
		Waiting list	0		
	Theatre	Education	0		
		Exercise	0		
		Music appreciation	0		
		Painting, dancing or tai chi	0		
		Physiotherapy	2	Mirabella et al [43]<br>Modugno et al [44]	Modugno et al [44] report standard error, which we need to convert to SD
		Support group	0		
		Usual care	0		
		Waiting list	0		
TUG	PD-specific dance	Education	0		
		Exercise	1	Hashimoto et al [56]	
		Music appreciation	0		
		Painting, dancing or tai chi	0		
		Physiotherapy	0		
		Support group	1	Michels et al [63-64]	
		Usual care	2	Hashimoto et al [56]<br>Ventura et al [77]	
		Waiting list	0		
	Tango-based dance	Education	1	De Natale et al [53]	
		Exercise	2	Hackney et al [54-55]<br>Romenets et al [58]	
		Music appreciation	0		
		Painting, dancing or tai chi	0		
		Physiotherapy	0		

		Support group	0		
		Usual care	2	Duncan and Earhart [65]<br>Hackney and Earhart [68-70]	Duncan and Earhart [65] excluded as no mean +SD values reported at follow-up. Meta-analysis not feasible.
		Waiting list	0		
	Singing	Education	0		
		Exercise	0		
		Music appreciation	0		
		Painting, dancing or tai chi	0		
		Physiotherapy	0		
		Support group	0		
		Usual care	0		
		Waiting list	0		
	Music therapy	Education	0		
		Exercise	0		
		Music appreciation	0		
		Painting, dancing or tai chi	0		
		Physiotherapy	0		
		Support group	0		
		Usual care	2	Pantelyat et al [48]<br>Pohl et al [45]	Pantelyat et al [48] and Pohl et al [45] excluded as no mean + SD values reported at follow-up – no meta-analysis feasible
		Waiting list	0		
	Theatre	Education	0		
		Exercise	0		
		Music appreciation	0		

		Painting, dancing or tai chi	0		
		Physiotherapy	0		
		Support group	0		
		Usual care	0		
		Waiting list	0		
MMSE	PD-specific dance	Education	0		
		Exercise	0		
		Music appreciation	0		
		Painting, dancing or tai chi	0		
		Physiotherapy	0		
		Support group	0		
		Usual care	0		
		Waiting list	0		
	Tango-based dance	Education	0		
		Exercise	0		
		Music appreciation	0		
		Painting, dancing or tai chi	0		
		Physiotherapy	0		
		Support group	0		
		Usual care	0		
		Waiting list	0		
	Singing	Education	0		
		Exercise	0		
		Music appreciation	0		
		Painting, dancing or tai chi	0		
		Physiotherapy	0		
		Support group	0		
		Usual care	0		

		Waiting list	0		
	Music therapy	Education	0		
		Exercise	0		
		Music appreciation	0		
		Painting, dancing or tai chi	0		
		Physiotherapy	0		
		Support group	0		
		Usual care	0		
		Waiting list	0		
	Theatre	Education	0		
		Exercise	0		
		Music appreciation	0		
		Painting, dancing or tai chi	0		
		Physiotherapy	0		
		Support group	0		
		Usual care	0		
		Waiting list	0		
FAB	PD-specific dance	Education	0		
		Exercise	1	Hashimoto et al [56]	
		Music appreciation	0		
		Painting, dancing or tai chi	0		
		Physiotherapy	0		
		Support group	0		
		Usual care	1	Hashimoto et al [56]	
		Waiting list	0		
	Tango-based dance	Education	1	De Natale et al [53]	
		Exercise	0		
		Music appreciation	0		

		Painting, dancing or tai chi	0		
		Physiotherapy	0		
		Support group	0		
		Usual care	0		
		Waiting list	0		
	Singing	Education	0		
		Exercise	0		
		Music appreciation	0		
		Painting, dancing or tai chi	0		
		Physiotherapy	0		
		Support group	0		
		Usual care	0		
		Waiting list	0		
	Music therapy	Education	0		
		Exercise	0		
		Music appreciation	0		
		Painting, dancing or tai chi	0		
		Physiotherapy	0		
		Support group	0		
		Usual care	1	Spina et al [46]	
		Waiting list	0		
	Theatre	Education	0		
		Exercise	0		
		Music appreciation	0		
		Painting, dancing or tai chi	0		
		Physiotherapy	0		
		Support group	0		
		Usual care	0		

		Waiting list	0		
MoCA	PD-specific dance	Education	0		
		Exercise	0		
		Music appreciation	0		
		Painting, dancing or tai chi	0		
		Physiotherapy	0		
		Support group	1	Michels et al [63-64]	
		Usual care	0		
		Waiting list	0		
	Tango-based dance	Education	1	McKee and Hackney [61]	
		Exercise	0		
		Music appreciation	0		
		Painting, dancing or tai chi	0		
		Physiotherapy	0		
		Support group	0		
		Usual care	0		
		Waiting list	0		
	Singing	Education	0		
		Exercise	0		
		Music appreciation	0		
		Painting, dancing or tai chi	0		
		Physiotherapy	0		
		Support group	0		
		Usual care	0		
		Waiting list	0		
	Music therapy	Education	0		
		Exercise	0		
		Music appreciation	0		

		Painting, dancing or tai chi	0		
		Physiotherapy	0		
		Support group	0		
		Usual care	1	Pantelyat et al [48]	
		Waiting list	0		
	Theatre	Education	0		
		Exercise	0		
		Music appreciation	0		
		Painting, dancing or tai chi	0		
		Physiotherapy	0		
		Support group	0		
		Usual care	0		
		Waiting list	0		
PDQ-39	PD-specific dance	Education	0		
		Exercise	0		
		Music appreciation	0		
		Painting, dancing or tai chi	0		
		Physiotherapy	0		
		Support group	1	Michels et al [63-64]	
		Usual care	2	Kalyani et al [72]<br>Ventura et al [77]	
		Waiting list	0		
	Tango-based dance	Education	1	Hackney et al [60]	
		Exercise	2	Rawson et al [57]<br>Romenets et al [58]	Rawson et al [57] excluded as no mean +SD values reported at follow-up – meta-analysis not feasible
		Music appreciation	0		

		Painting, dancing or tai chi	0		
		Physiotherapy	0		
		Support group	0		
		Usual care	1	Hackney et al [68-70]	
		Waiting list			
	Singing	Education	0		
		Exercise	0		
		Music appreciation	1	Matthews et al [51]	
		Painting, dancing or tai chi	0		
		Physiotherapy	0		
		Support group	0		
		Usual care	0		
		Waiting list	0		
	Music therapy	Education	0		
		Exercise	0		
		Music appreciation	0		
		Painting, dancing or tai chi	0		
		Physiotherapy	1	Pacchetti et al [47]	
		Support group	0		
		Usual care	3	Pantelyat et al [48]<br>Pohl et al [45]<br>Spina et al [46]	Pantelyat et al [48] and Pohl et al [45] excluded as no mean + SD values reported at follow-up – meta-analysis not feasible
		Waiting list	0		
	Theatre	Education	0		
		Exercise	0		
		Music appreciation	0		

		Painting, dancing or tai chi	0		
		Physiotherapy	2	Mirabella et al [43]<br>Modugno et al [44]	Modugno et al [44] excluded as no PDQ-39 total score reported (only subscales) – meta-analysis not feasible
		Support group	0		
		Usual care	0		
		Waiting list	0		

There were 6 feasible meta-analysis sets:

1. UPDRS motor for tango-based dance vs exercise (3 studies; De Natale et al [53]; Hackney et al [54-55]; Romenets et al [58])
2. UPDRS motor for tango-based dance vs usual care (2 studies; Duncan and Earhart [66] /Foster et al [67]; Hackney and Earhart [68-70])
3. UPDRS motor for theatre vs physiotherapy (2 studies; Mirabella et al [43]; Modugno et al [44])
4. TUG for PD-specific dance vs usual care (2 studies; Hashimoto et al [56]; Ventura et al [77])
5. TUG for tango-based dance vs exercise (2 studies; Hackney et al [54-55]; Romenets et al [58])
6. PDQ-39 for PD-specific dance vs usual care (2 studies; Kalyani et al [72]; Ventura et al [77]) – can do using change scores too for this set.

In total, there were 10 studies that could contribute data to the meta-analysis.

Part B. **Tabulation of data**

Set 1: UPDRS motor for tango-based dance vs exercise

**Study**	**Intervention**			**Control**		
	N	Mean	SD	N	Mean	SD
De Natale et al [53]	9[a]	16.12	7.55	7[a]	14	9.9
Hackney et al [54-55]	9	22.6	1.3	10	20.6	1.2
Romenets et al [58]	18[b]	19.1	10.2	15[b]	26.3	13.5

Comparison conducted on follow-up scores. [a] = using headline N – 2 participants dropped out, but it is not stated from which arm(s). [b] = the primary analysis was intention to treat, though there were 9 protocol violations, of which 7 occurred in the intervention arm. N = number, SD = standard deviation.

Set 2: UPDRS motor for tango-based dance vs usual care

Study	Intervention			Control		
	N	Mean	SD	N	Mean	SD
Duncan and Earhart [66] /Foster et al [67]	26	31.7	2.4	26	45.0	1.9
Hackney and Earhart [68-70]	14	26.0	2.5	17	32.4	2.6

Comparison conducted on follow-up scores.

Set 3: UPDRS motor for theatre vs physiotherapy

Study	Intervention			Control		
	N	Mean	SD	N	Mean	SD
Mirabella et al [43]	12	24.2	9.9	12	22	4.9
Modugno et al [44]	10	19.5	10.53[a]	10	21.7	12.74[a]

Comparison conducted on follow-up scores. [a] = converted from standard error.

Set 4: TUG for PD-specific dance vs usual care

Study	Intervention			Control		
	N	Mean	SD	N	Mean	SD
Hashimoto et al [56]	15	9.7	2.1	14	10.2	2.4
Ventura et al [77]	8	11.3	1.9	7	16.3	6.5

Comparison conducted on follow-up scores. Time data used for TUG.

Set 5: TUG for tango-based dance vs exercise

Study	Intervention			Control		
	N	Mean	SD	N	Mean	SD
Hackney et al [54-55]	9	9.8	0.4	10	11.8	0.4
Romenets et al [58]	18[a]	6.1	1.5	15[a]	8.0	2.2

Comparison conducted on follow-up scores. Time data used for TUG. a = the primary analysis was intention to treat, though there were 9 protocol violations, of which 7 occurred in the intervention arm.

Set 6: PDQ-39 for PD-specific dance vs usual care

Study	Intervention			Control		
	N	Mean	SD	N	Mean	SD

Kalyani et al [72]	17	-4.74	6.76[a]	16	2.07	5.95[a]
Ventura et al [77]	8	-8.1[b]	7.4	7	4.0[c]	10.4

Comparison conducted on change scores.. [a] = converted from 95% confidence interval for input into meta-analysis. b = presented by authors as a positive value as represents an improvement, but is numerically a reduction in score, and needs to be entered as a negative value in meta-analysis. c = presented by authors as a negative value as represents a deterioration, but it is numerically an increased in score, and needs to be entered as a positive value in meta-analysis.

Part C. **Meta-analysis forest plots**

	Tango-based dance			Exercise				Mean Difference
**Study or Subgroup**	**Mean**	**SD**	**Total**	**Mean**	**SD**	**Total**	**Weight**	**IV, Random, 95% CI**
De Natale et al [53]	16.12	7.55	9	14	9.9	7	21.8%	2.12 [-6.72, 10.96]
Romenets et al [58]	19.1	10.2	18	26.3	13.5	15	23.5%	-7.20 [-15.50, 1.10]
Hackney et al [54-55]	22.6	1.3	9	20.6	1.2	10	54.7%	2.00 [0.87, 3.13]
**Total (95% CI)**			**36**			**32**	**100.0%**	**-0.13 [-5.41, 5.14]**

Heterogeneity: Tau² = 12.90; Chi² = 4.64, df = 2 (P = 0.10); I² = 57%
Test for overall effect: Z = 0.05 (P = 0.96)

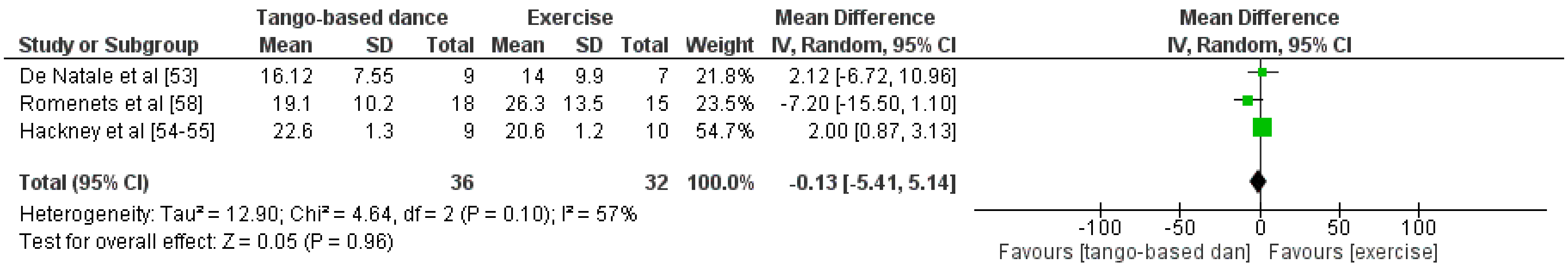

Caption

Forest plot for set 1: UPDRS motor for tango-based dance vs exercise

	Tango-based dance			Usual care				Mean Difference
**Study or Subgroup**	**Mean**	**SD**	**Total**	**Mean**	**SD**	**Total**	**Weight**	**IV, Random, 95% CI**
Hackney and Earhart [68-70]	26	2.5	14	32.4	2.6	14	49.4%	-6.40 [-8.29, -4.51]
Duncan and Earhart [66]/Foster et al [67]	31.7	2.4	26	45	1.9	26	50.6%	-13.30 [-14.48, -12.12]
**Total (95% CI)**			**40**			**40**	**100.0%**	**-9.89 [-16.65, -3.13]**

Heterogeneity: Tau² = 23.16; Chi² = 36.92, df = 1 (P < 0.00001); I² = 97%
Test for overall effect: Z = 2.87 (P = 0.004)

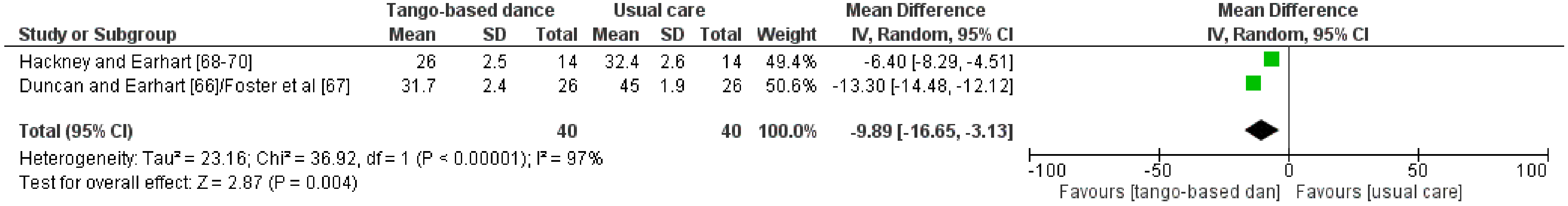

Caption

Forest plot for set 2: UPDRS motor for tango-based dance vs usual care

Study or Subgroup	Theatre Mean	Theatre SD	Theatre Total	Physiotherapy Mean	Physiotherapy SD	Physiotherapy Total	Weight	Mean Difference IV, Random, 95% CI
Modugno et al [44]	19.5	10.53	10	21.7	12.74	10	27.1%	-2.20 [-12.44, 8.04]
Mirabella et al [43]	24.2	9.9	12	22	4.9	12	72.9%	2.20 [-4.05, 8.45]
**Total (95% CI)**			**22**			**22**	**100.0%**	**1.01 [-4.33, 6.34]**

Heterogeneity: Tau² = 0.00; Chi² = 0.52, df = 1 (P = 0.47); I² = 0%

Test for overall effect: Z = 0.37 (P = 0.71)

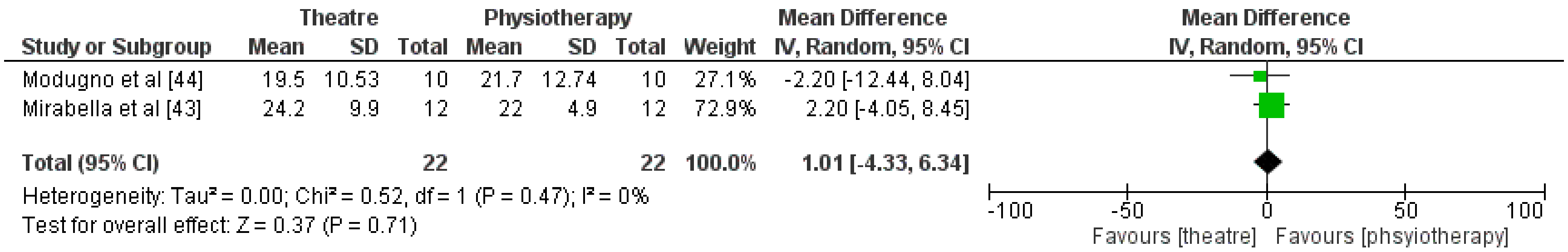

Caption

Forest plot for set 3: UPDRS motor for theatre vs physiotherapy

Study or Subgroup	PD-specific dance Mean	PD-specific dance SD	PD-specific dance Total	Usual care Mean	Usual care SD	Usual care Total	Weight	Mean Difference IV, Random, 95% CI
Ventura et al [77]	11.3	1.9	8	16.3	6.5	7	35.7%	-5.00 [-9.99, -0.01]
Hashimoto et al [56]	9.7	2.1	15	10.2	2.4	14	64.3%	-0.50 [-2.15, 1.15]
**Total (95% CI)**			**23**			**21**	**100.0%**	**-2.11 [-6.33, 2.12]**

Heterogeneity: Tau² = 6.53; Chi² = 2.82, df = 1 (P = 0.09); I² = 64%

Test for overall effect: Z = 0.98 (P = 0.33)

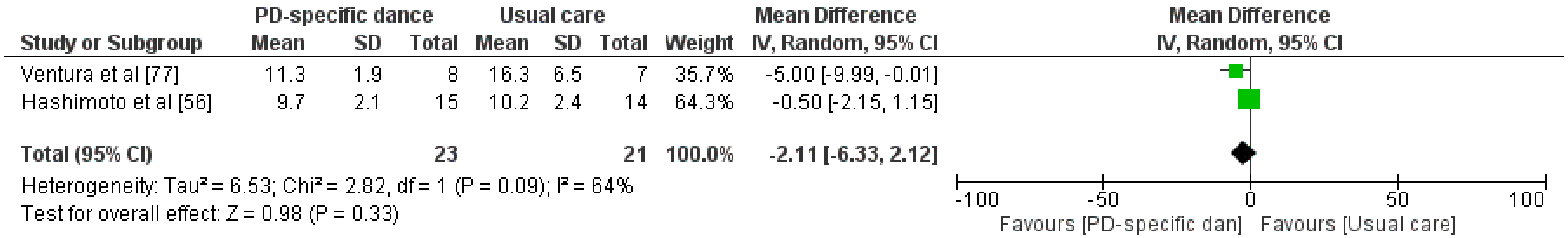

Caption

Forest plot for set 4: TUG for PD-specific dance vs usual care

Study or Subgroup	Tango-based dance Mean	SD	Total	Exercise Mean	SD	Total	Weight	Mean Difference IV, Random, 95% CI
Romenets et al [58]	6.1	1.5	18	8	2.2	15	7.0%	-1.90 [-3.21, -0.59]
Hackney et al [54-55]	9.8	0.4	9	11.8	0.4	10	93.0%	-2.00 [-2.36, -1.64]
**Total (95% CI)**			**27**			**25**	**100.0%**	**-1.99 [-2.34, -1.65]**

Heterogeneity: Tau² = 0.00; Chi² = 0.02, df = 1 (P = 0.89); I² = 0%
Test for overall effect: Z = 11.25 (P < 0.00001)

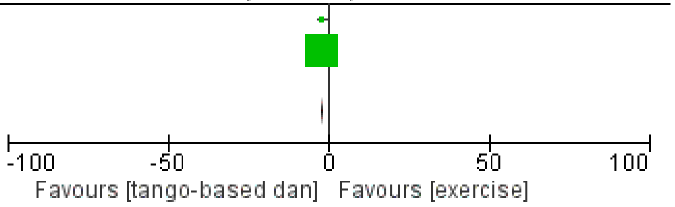

Caption

Forest plot for set 5: TUG for tango-based dance vs exercise

Study or Subgroup	PD-specific dance Mean	SD	Total	Usual care Mean	SD	Total	Weight	Mean Difference IV, Random, 95% CI
Ventura et al [77]	-8.1	7.4	8	4	10.4	7	18.9%	-12.10 [-21.35, -2.85]
Kalyani et al [72]	-4.74	6.76	17	2.07	5.95	16	81.1%	-6.81 [-11.15, -2.47]
**Total (95% CI)**			**25**			**23**	**100.0%**	**-7.81 [-11.87, -3.75]**

Heterogeneity: Tau² = 0.39; Chi² = 1.03, df = 1 (P = 0.31); I² = 3%
Test for overall effect: Z = 3.77 (P = 0.0002)

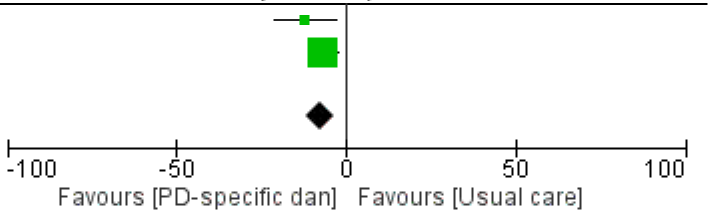

Caption

Forest plot for set 6: PDQ-39 for PD-specific dance vs usual care (change score analysis)

Appendix 8. **Full list of included studies**

Allen, J.L., McKay, J.L., Sawers, A., Hackney, M.E., & Ting, L.H. (2017). Increased neuromuscular consistency in gait and balance after partnered, dance-based rehabilitation in Parkinson's disease. *Journal of Neurophysiology*, 118, 363-373.

Azekawa, M., & Lagasse, A.B. (2018). Singing exercises for speech and vocal abilities in individuals with hypokinetic dysarthria: a feasibility study. *Music Therapy Perspectives*, 36, 40-49.

Batson, G., Migliarese, S.J., Soriano, C., Burdette, J.H., & Laurienti, P.J. (2014). Effects of improvisational dance on balance in Parkinson's disease: a two-phase fMRI case study. *Physical and Occupational Therapy in Geriatrics*, 2014, 32 (3), 188-197.

Batson, G. (2010). Feasibility of an intensive trial of modern dance for adults with Parkinson disease. *Complementary Health Practice Review*, 15(2), 65-83.

Bearss, K.A., McDonald, K.C., Bar, R.J., & DeSouza, J.F.X. (2017). Improvements in balance and gait speed after a 12 week dance intervention for Parkinson's disease. *Archives in Integrative Medicine*, 4, 10-13.

Blandy, L.M., Beevers, W.A., Fitzmaurice, K., & Morris, M.E. (2015). Therapeutic Argentine tango dancing for people with mild Parkinson's disease: a feasibility study. *Frontiers in Neurology*, 6, 122.

Clifford, M., Robey, S., & Dixon, J. (2017). Dancing with Parkinson's: a London hospice's experience of running a dance programme. *International Journal of Palliative Nursing*, 23, 498-500.

De Natale, E.R., Paulus, K.S., Aiello, E., Sanna, B., Manca, A., Sotgiu, G., Leali, P.T., & Deriu, F. (2017). Dance therapy improves motor and cognitive functions in patients with Parkinson's disease. *NeuroRehabilitation*, 40, 141-144.

Di Benedetto, P., Cavazzon, M., Mondolo, F., Rugiu, G., Peratoner, A., & Biasutti, E. (2009).Voice and choral singing treatment: A new approach for speech and voice disorders in Parkinson's disease. *European Journal of Physical and Rehabilitation Medicine*, 45, 13-19.

Duncan, R.P., & Earhart, G.M. (2014). Are the effects of community-based dance on Parkinson disease severity, balance, and functional mobility reduced with time? A 2-year prospective pilot study. *Journal of Alternative and Complementary Medicine*, 20, 757-763.

Duncan, R.P., & Earhart, G.M. (2012). Randomized controlled trial of community-based dancing to modify disease progression in Parkinson disease. *Neurorehabilitation and Neural Repair*, 26(2), 132-143.

Elefant, C., Baker, F.A., Lotan, M., Lagesen, S.K., & Skeie, G.O. (2012). The effect of group music therapy on mood, speech, and singing in individuals with Parkinson's disease – a feasibility study. *Journal of Music Therapy*, 49, 278-302.

Elefant, C., Lotan, M., Baker, F.A., & Skeie, G.O. (2012). Effects of music therapy on facial expression of individuals with Parkinson's disease: a pilot study. *Musicae Scientae*, 13, 392-400.

Evans, C., Canavan, M., Foy, C., Langford, R., & Proctor, R. (2012). Can group singing provide effective speech therapy for people with Parkinson's disease? *Arts & Health*, 4, 83-95.

Foster, E.R., Golden, L., Duncan, R.P., & Earhart, G.M. (2013). A community-based Argentine tango dance program is associated with increased activity participation among individuals with Parkinson disease. *Archives of Physical and Medical Rehabilitation*, 94(2), 240-249.

Hackney, M.E., Kantorovich, S., & Earhart, G.M. (2007). A study of the effects of Argentine tango as a form of partnered dance for those with Parkinson disease and the healthy elderly. *American Journal of Dance Therapy*, 29(2), 109-127.

Hackney, M.E., Kantorovich, S., Levin, R., & Earhart, G.M. (2007). Effects of tango on functional mobility in Parkinson's disease: a preliminary study. *Journal of Neurologic Physical Therapy*, 31, 173-179.

Hackney, M.E., & Earhart, G.M. (2009). Effects of dance on movement control in Parkinson's disease: a comparison of Argentine tango and American ballroom. *Journal of Rehabilitative Medicine*, 41, 475-481.

Hackney, M.E., & Earhart, G.M. (2009). Health-related quality of life and alternative forms of exercise in Parkinson disease. *Parkinsonism and Related Disorders*, 15, 644-648.

Hackney, M.E., & Earhart, G.M. (2009). Short duration, intensive tango dancing for Parkinson disease: an uncontrolled pilot study. *Complementary Therapies in Medicine*, 17(4), 203-207.

Hackney, M.E., & Earhart, G.M. (2010). Effects of dance on gait and balance in Parkinson disease: a comparison of partnered and non-partnered dance movement. *Neurorehabilitation and Neural Repair*, 24, 384-392.

Hackney, M., Hart, A., Kim, C. (2018). It takes two to tango but following may be more beneficial for rehabilitating people with mild-moderate Parkinson's [Abstract]. *Movement Disorders*, 33 (S2), S149.

Hashimoto, H., Takabatake, S., Miyaguchi, H., Nakanishi, H., & Naitou, Y. (2015). Effects of dance on motor functions, cognitive functions, and mental symptoms of Parkinson's disease: a quasi-randomized pilot trial. *Complementary Therapies in Medicine*, 23, 210-219.

Heiberger, L., Maurer, C., Amtage, F., Mendez-Balbuena, I., Schulte-Mönting, J., Hepp-Raymond, M-C., & Kristeva, R. (2011). Impact of a weekly dance class on the functional mobility and on the quality of life of individuals with Parkinson's disease. *Frontiers in Aging Neuroscience*, 3, 14.

Higgins, A.N., & Richardson, K.C. (2019). The effects of a choral singing intervention on speech characteristics in individuals with Parkinson's disease: an exploratory study. *Communication Disorders Quarterly*, 40(4), 195-205.

Hulbert, S., Ashburn, A., Roberts, L., & Verheyden, G. (2017). Dance for Parkinson's – the effects on whole body co-ordination during turning around. *Complementary Therapies in Medicine*, 32, 91-97.

Irons, J.Y., Hancox, G., Vella-Burrows, T., Han, E-Y., Chong, H-J., Sheffield, D., & Stewart, D.E. (2020). Group singing improves quality of life for people with Parkinson's: an international study, *Aging and Mental Health*, https://doi.org/10.1080/13607863.2020.1720599.

Irons, Y., Hancox, G., Vella-Burrows, T., Han, E-Y., Ching, H-J, Sheffield, D., & Stewart, D. (2019). Group singing improves quality of life in people with Parkinson's: an international Sing to Beat Parkinson's project [Abstract]. *Journal of Parkinson's Disease*, 9, 186.

Kalyani, H.H.N., Sullivan, K.A., Moyle, G., Brauer, S., Jeffrey, E.R, & Kerr, G.K. (2019). Impacts of dance on cognition, psychological symptoms and quality of life in Parkinson's disease. *NeuroRehabilitation,* 45, 273-283.

Koch, S.C., Mergheim, K., Raeke, J., Machado, C.B., Riegner, E., Nolden, J…Hillecke, T.K. (2016). The embodied self in Parkinson's disease: feasibility of a single tango intervention for assessing changes in psychological health outcomes and aesthetic experience. *Frontiers in Neuroscience*, 10, 287.

Kunkel, D., Fitton, C., Roberts, L., Pickering, R.M., Roberts, H.C., Wiles, R…Ashburn, A. (2017). A randomized controlled feasibility trial exploring partnered ballroom dancing for people with Parkinson's disease. *Clinical Rehabilitation*, 31, 1340-1350.

Lee, H-J., Kim, S-Y., Chae, Y., Kim, M-Y., Yin, C., Jung, W-S…Lee, H. (2018). Turo (Qi dance) program for Parkinson's disease patients: randomized, assessor blind, waiting-list control, partial crossover study. *Explore,* 14, 216-223.

McGill, A., Houston, S., & Lee, R.Y.W. (2019). Effects of a ballet-based dance intervention on gait variability and balance confidence of people with Parkinson's. *Arts and Health*, 11(2): 133-146.

McKay, J.L., Ting, L.H., & Hackney, M.E. (2016). Balance, body motion and muscle activity after high volume short term dance-based rehabilitation in individuals with Parkinson's disease: a pilot study. *Journal of Neurologic Physical Therapy*, 40, 257-268.

McKee, K.E., & Hackney, M.E. (2013). The effects of adapted tango on spatial cognition and disease severity in Parkinson's disease. *Journal of Motor Behavior*, 45(6), doi:10.1080/00222895.2013.834288.

McNeely, M.E., Mai, M.M., Duncan, R,P., & Earhart, G.M. (2015). Differential effects of tango versus Dance for PD in Parkinson disease. *Frontiers in Aging Neuroscience*, 7, 239.

McRae, C., Leventhal, D., Westheimer, O., Mastin, T., Utley, J., & Russell, D. (2018). Long-term effects of Dance for PD on self-efficacy among persons with Parkinson's disease. *Arts & Health,* 10(1), 85-96.

Marchant, D., Sylvester, J.L., & Earhart, G.M. (2010). Effects of a short duration, high dose contact improvisation dance workshop on Parkinson disease: a pilot study. *Complementary Therapies in Medicine,* 18, 184-190.

Matthews, R., Purdy, S., & Tippett, L. (2018). Acoustic, respiratory, cognitive and wellbeing comparisons of two groups of people with Parkinson's disease participating in voice and choral singing group therapy (VCST) versus music appreciation activity [Abstract]. *Movement Disorders*, 33 (S2), S406.

Michels, K., Dubaz, O., Hornthal, E., & Bega, D. (2018). "Dance Therapy" as a psychotherapeutic movement intervention in Parkinson's disease. *Complementary Therapies in Medicine*, 40, 248-252.

Michels, K., Hornthal, E., & Bega, D. (2018). A pilot study on dance/movement therapy in Parkinson's disease [Abstract]. *Neurology,* 90 (15, S1).

Mirabella, G., De Vita, P., Fragola, M., Rampelli, S., Lena, F…Modugno, N. (2017). Theatre is a valid add-on therapeutic intervention for emotional rehabilitation of Parkinson's disease patients. *Parkinson's Disease*, 2017, 7436725.

Modugno, N., Iaconelli, S., Fiorelli, M., Lena, F., Kusch, I., & Mirabella, G. (2010). Active theater as a complementary therapy for Parkinson's disease rehabilitation: a pilot study. *The Scientific World Journal*, 10, 2301-2313.

Pacchetti, C., Mancini, F., Aglieri, R., Fundarò, C., Martignoni, E.,& Nappi, G. (2000). Active music therapy in Parkinson's disease: an integrative method for motor and emotional rehabilitation. *Psychosomatic Medicine*, 62, 386-393.

Pantelyat, A., Syres, C., Reichwein, S., & Willis, A. (2016). DRUM-PD: the use of a drum circle to improve the symptoms and signs of Parkinson's disease (PD). *Movement Disorders Clinical Practice*, 3, 243-249.

Patel, R., Hart, A., Hackney, M., & Kim, C. (2018). Partnered, rhythmic, rehabilitative movement may impact medication related motor fluctuations [Abstract]. *Annals of Neurology*, 84 (S22), S201.

Pohl, P., Didzar, N., & Hallert, E. (2013). The Ronnie Gardiner Rhythm and Music Method – a feasibility study in Parkinson's disease. *Disability and Rehabilitation*, 35, 2197-2204.

Prewitt, C.M., Charpentier, J.C., Brosky, J.A., & Urbscheit, N.L. (2017). Effects of dance classes on cognition, depression, and self-efficacy in Parkinson's disease. *American Journal of Dance Therapy*, 39, 126-141.

Rawson, K.S., McNeely, M.E., Duncan, R.P., Pickett, K.A., Perlmutter, J.S., & Earhart, G.M. (2019). Exercise and Parkinson's disease: comparing tango, treadmill and stretching. *Journal of Neurologic Physical Therapy*, 43(1), 26-32.

Rocha, P., Aguiar, L., McClelland, J.A., & Morris, M.E. (2018). Dance therapy for Parkinson's disease: a randomised feasibility trial. *International Journal of Therapy and Rehabilitation*, 25 (2), 65-72.

Romenets, S.R., Anang, J., Fereshtehnejad, S-M., Pelletier, A., & Postuma, R. (2015). Tango for treatment of motor and non-motor manifestations in Parkinson's disease: a randomized control study. *Complementary Therapies in Medicine*, 23, 175-184.

Shanahan, J., Morris, M.E., Ni Bhriain, O., Volpe, D., Lynch, T., & Clifford, A. (2017). Dancing for Parkinson's disease: a randomized controlled trial of Irish set dancing compared with usual care. *Archives of Physical Medicine and Rehabilitation*, 98, 1744-1751.

Shanahan, J., Morris, M.E., Ni Bhriain, O., Volpe, D., Richardson, M., & Clifford, A.M. (2015). Is Irish set dancing feasible for people with Parkinson's disease in Ireland? *Complementary Therapies in Medicine*, 21, 47-51.

Shih, L.C., Piel, J., Warren, A., Kraiscs, L., Silver, A., Vanderhorst, V., … Tarsy, D. (2012). Singing in groups for Parkinson's disease (SING-PD): A pilot study of group singing therapy for PD-related voice/speech disorders. *Parkinsonism and Related Disorders*, 18, 548-552.

Solla, P., Cugusi, L., Bertoli, M., Cereatti, A., Della Croce, U., Pani, D…Mercuro, G. (2019). Sardinian folk dance for individuals with Parkinson's disease: a randomized

controlled pilot trial. *Journal of Alternative and Complementary Medicine*, 25, 305-316.

Spina, E., Barone, P., Mosca, L.L., Lombardi, A., Longo, K., Iavarone, A., & Amboni, M. (2016). Music therapy for motor and nonmotor symptoms of Parkinson's disease: a prospective, randomized, controlled, single-blinded study. *Journal of the American Geriatrics Society*, 64 (9), e36-e38.

Stegemöller, E.L., Hibbing, P., Radig, H., & Wingate, J. (2017). Therapeutic singing as an early intervention for swallowing in persons with Parkinson's disease. *Complementary Therapies in Medicine*, 31, 127-133.

Stegemöller, E.L., Radig, H., Hibbing, P., Wingate, J., & Sapienza, C. (2017). Effects of singing on voice, respiratory control and quality of life in persons with Parkinson's disease. *Disability and Rehabilitation*, 39, 594-600.

Tamplin, J., Morris, M.E., Marigliani, C., Baker, F.A., Vogel, A.P. (2019). ParkinSong: a controlled trial of singing-based therapy for Parkinson's disease. *Neurorehabilitation and Neural Repair*, 33, 453-463.

Tamplin, J., Vogel, A., Marigliani, C., Baker, F., Morris, M. (2018). A controlled trial of ParkinSong singing groups to improve communication and wellbeing in Parkinson's disease [Abstract]. *Movement Disorders*, 33 (S2), S138.

Tanner, M., Rammage, L., & Liu, L. (2016). Does singing and vocal strengthening improve vocal ability in people with Parkinson's disease? *Arts & Health*, 8(3), 199-212.

Ventura, M.I., Barnes, D.E., Ross, J.M., Lanni, K.E., Sigvardt, K.A,, & Disbrow, E.A. (2016). A pilot study to evaluate multi-dimensional effects of dance for people with Parkinson's disease. *Contemporary Clinical Trials*, 51, 50-55.

Volpe, D., Signorini, M., Marchetto, A., Lynch, T., & Morris, M.E. (2013). A comparison of Irish set dancing and exercises for people with Parkinson's disease: a phase II feasibility study. *BMC Geriatrics*, 13, 54.

Westbrook, B.K., & McKibben, H. (1989). Dance/movement therapy with groups of outpatients with Parkinson's disease. *American Journal of Dance Therapy*, 11(1), 27-38.

Westheimer, O., McRae, C., Henchcliffe, C., Fesharaki, A., Glazman, S., Ene, H., & Bodis-Wollner, I. (2015). Dance for PD: a preliminary investigation of effects on motor function and quality of life among persons with Parkinson's disease (PD). *Journal of Neural Transmission*, 122, 1263-1270.

Yinger, O.S., & Lapointe. L.L. (2012). The effects of participation in a group music therapy voice protocol (G-MTVP) on the speech of individuals with Parkinson's disease. *Music Therapy Perspectives*, 30, 25-31.

Zafar, M., Bozzorg, A., & Hackney, M.E. (2017). Adapted tango improves aspects of participation in older adults versus individuals with Parkinson's disease. *Disability and Rehabilitation*, 39, 2294-2301.
